# Intertwining DNA-RNA nanocapsules loaded with tumor neoantigens as synergistic nanovaccines for cancer immunotherapy

**DOI:** 10.1038/s41467-017-01386-7

**Published:** 2017-11-14

**Authors:** Guizhi Zhu, Lei Mei, Harshad D. Vishwasrao, Orit Jacobson, Zhantong Wang, Yijing Liu, Bryant C. Yung, Xiao Fu, Albert Jin, Gang Niu, Qin Wang, Fuwu Zhang, Hari Shroff, Xiaoyuan Chen

**Affiliations:** 1 Laboratory of Molecular Imaging and Nanomedicine, National Institute of Biomedical Imaging and Bioengineering (NIBIB), National Institutes of Health (NIH), Bethesda, MD 20892 USA; 2 Department of Nutrition and Food Science, College of Agriculture and Natural Resources, University of Maryland, College Park, MD 20742 USA; 3 Advanced Imaging and Microscopy Resource, NIH, Bethesda, MD 20892 USA; 4 Laboratory of Cellular Imaging and Macromolecular Biophysics, NIBIB, NIH, Bethesda, MD 20892 USA; 5 Section on High Resolution Optical Imaging, NIBIB, NIH, Bethesda, MD 20892 USA

## Abstract

Nanomedicines that co-deliver DNA, RNA, and peptide therapeutics are highly desirable yet remain underdeveloped for cancer theranostics. Herein, we report self-assembled intertwining DNA-RNA nanocapsules (iDR-NCs) that efficiently delivered synergistic DNA CpG and short hairpin RNA (shRNA) adjuvants, as well as tumor-specific peptide neoantigens into antigen presenting cells (APCs) in lymph nodes for cancer immunotherapy. These nanovaccines were prepared by (1) producing tandem CpG and shRNA via concurrent rolling circle replication and rolling circle transcription, (2) self-assembling CpG and shRNA into DNA-RNA microflowers, (3) shrinking microflowers into iDR-NCs using PEG-grafted cationic polypeptides, and (4) physically loading neoantigen into iDR-NCs. CpG and shRNA in iDR-NCs synergistically activate APCs for sustained antigen presentation. Remarkably, iDR-NC/neoantigen nanovaccines elicit 8-fold more frequent neoantigen-specific peripheral CD8^+^ T cells than CpG, induce T cell memory, and significantly inhibit the progression of neoantigen-specific colorectal tumors. Collectively, iDR-NCs represent potential DNA/RNA/peptide triple-co-delivery nanocarriers and synergistic tumor immunotherapeutic nanovaccines.

## Abstract (web summary)

Nucleic acid nanomedicines are promising for cancer drug delivery. Here, the authors show using a mouse model the tumor immunotherapeutic efficacy of nanovaccines based on intertwining DNA-RNA nanocapsules loaded with DNA CpG, *Stat3*-silencing short hairpin RNA and tumor-specific peptide neoantigens.

## Introduction

Natural nucleic acids (e.g., DNA, RNA) have inspired the development of nucleic acid nanotechnology and synthetic functional nucleic acids. Specifically, inspired by the abilities of natural nucleic acids to structurally code and store genetic information, nucleic acid nanotechnology utilizes synthetic nucleic acids to precisely engineer nanostructures^1–5^. Nucleic acid nanotechnology has enabled the development of various nucleic acid nanomedicines for biomedical applications, including drug delivery^3–7^. Further, inspired by the biological functions of natural nucleic acids, synthetic functional nucleic acids have been developed as therapeutics^8,9^ and targeting ligands^10,11^. For instance, unmethylated cytosine–guanine oligodeoxynucleotides (CpG) have been extensively investigated as immunomodulatory cancer therapeutics^8,12^. CpG triggers the Toll-like receptor 9 (TLR9) pathway to activate APCs, such as dendritic cells (DCs) and macrophages^13^. In addition, RNA therapeutics, such as shRNA, are another class of nucleic acid therapeutics that precisely regulate gene expression via RNA interference^14^. Empowered by the inherent structure programmability and versatile functionalities of nucleic acids, nucleic acid nanotechnology enables engineering of multiple functional nucleic acids into a single nanomedicine, which is especially of interest in co-delivering multiple synergistic nucleic acid therapeutics for combination cancer therapy. Towards this end, a simple technology to construct nucleic acid nanostructures that incorporate both DNA and RNA therapeutics is highly desirable, yet remains an underdeveloped field of study. Previously, nucleic acid nanostructures have been constructed *via* nucleic acid hybridization^7^, and recently via co-crystallization of nucleic acids with inorganic supplements during rolling circle replication (RCR) and rolling circle transcription (RCT) to respectively construct DNA^5,15^ or RNA^6,16,17^ nanostructures. However, these technologies are either limited by complicated design, limited biostability, or most importantly, the lack of ability to simply incorporate both DNA and RNA therapeutics into the same nanomedicine for co-delivery.

Cancer immunotherapy has made encouraging breakthroughs in the past decades, which modulates the immune system to treat cancer^18^. Because the immune system in cancer patients is compromised by multi-tier immunosuppressive signaling pathways^19^, combination therapy is especially significant for cancer immunotherapy by combining synergistic therapeutics to modulate multiple pathways, *via*, for example, activating anticancer immunostimulatory pathways and deactivating immunosuppressive pathways. As mentioned, CpG has shown encouraging clinical efficacy in cancer treatment by activating the immunostimulatory TLR9 pathway^20^. Moreover, Janus kinase (JAK)–signal transducer and activator of transcription (STAT) pathways have been shown as promising targets for cancer immunotherapy^21^. STAT3, a member of the STAT family, is immunosuppressive in APCs by multiple mechanisms such as inducing antigen-specific T cell tolerance^22–24^. Even worse, STAT3 suppresses CpG-activated immunostimulation^25^, suggesting the clinical demand to synergistically activate TLR9 pathway and disarm STAT3 pathway in APCs for combination cancer immunotherapy^26–28^. Towards this end, therapeutic CpG and *Stat3* shRNA are promising, yet the clinical applications of these therapeutics have thus far been hindered by: (1) limited in vivo nucleic acid biostability (e.g., nuclease susceptibility) or otherwise costly chemical modification to improve biostability, (2) suboptimal pharmacodynamics and pharmacokinetics that result in rapid clearance and inefficient delivery in vivo, (3) inefficient intracellular nucleic acid delivery due to negative charges, (4) limited therapeutic efficacy of monotherapy, and (5) toxicity of many current nucleic acid delivery vehicles.

Nanovaccines, owing to typically efficient delivery into secondary lymphoid organs such as lymph nodes (LNs), have been enthusiastically explored for delivering immunomodulatory adjuvants and antigens for cancer immunotherapy^29–34^, which together can elicit potent and durable adaptive immunity. While previous investigation of tumor-associated antigens has yielded moderate therapeutic efficacy with risks of side effects associated with autoimmune responses, tumor-specific neoantigens have emerged as a promising alternative for potent and safe immunotherapy. Preclinical and clinical evidences have supported the key role of neoantigens in tumor immunotherapy^35–37^. Unlike tumor-associated antigens that are expressed in both tumors and healthy tissues, neoantigens, which are derived from somatic mutations in tumors, are expressed exclusively in tumor cells but not in healthy cells. Therefore, the use of neoantigens as vaccine components would potentially avoid autoimmunity against healthy tissues, and nanovaccines that co-deliver adjuvants and neoantigens are of tremendous interest for tumor immunotherapy^29,33^.

Herein, we developed biostable iDR-NC/neoantigen complexes as nanovaccines that incorporated CpG and *Stat3* shRNA, as well as tumor-specific neoantigens for efficient co-delivery and immunomodulation in cancer immunotherapy. Unlike DNA and RNA nanostructures that were previously constructed via separate RCR or RCT^5,16^, iDR-NCs are hybrid DNA-RNA nanostructures that are generated via combined RCR and RCT in the same reaction system, which essentially incorporates both DNA and RNA therapeutics into the single nanostructures. Moreover, given the large sizes of microstructures constructed from RCT or RCR (*d*: ~ 0.5 − 5 µm), it is essential to shrink them for efficient drug delivery. Although polyethylenimine (PEI) can meet this end, the cytotoxicity of highly cationic PEI raises serious safety concerns^16^. In this study, we synthesized biocompatible PEG-grafted polypeptide (PPT-*g*-PEG) copolymers to shrink the microflowers (MFs) constructed from combined RCR/RCT. Specifically, PPT-*g*-PEG imparts multiple functions to precisely engineer nanovaccine delivery: (1) the cationic PPTs transform MFs into NCs to enhance delivery efficiency to LNs at the tissue level and to APCs at the cellular level, (2) acid-labile PEG not only ensures high solubility of copolymer and thus effective MF shrinkage, but also enhances the proton sponge effect to promote intracellular delivery after PEG shedding and hence exposure of cationic PPTs in acidic endolysosomes, (3) the hydrophobic PPTs allows loading of tumor-specific neoantigens into iDR-NCs via hydrophobic interactions between peptide neoantigens and PPTs for co-delivery of adjuvants and antigens, and (4) biocompatible and biodegradable PPT-*g*-PEG is expected to have good safety profile. The resulting iDR-NCs synergistically activated APCs, and iDR-NC/neoantigen complexes elicited potent and durable tumor-specific antitumor immunity for tumor immunotherapy.

## Results

### Self-assembly of iDR-NCs as nanoadjuvants

**Fig. 1:**
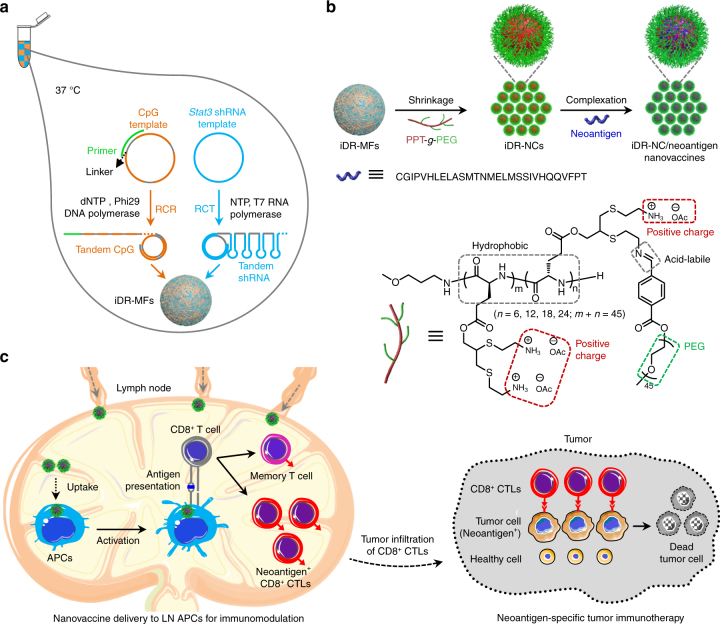
Schematics of iDR-NC/neoantigen nanovaccines for synergistic tumor immunotherapy. **a** Concurrent RCR and RCT in the same solution generated tandem CpG and *Stat3* shRNA, which were self-assembled into intertwining DNA-RNA MFs. **b** The above MFs were shrunk by PPT-*g*-PEG to form iDR-NCs, which was further loaded with tumor-specific neoantigen via hydrophobic interactions between peptide antigens and hydrophobic PPT moieties. **c** In immunocompetent mice, iDR-NCs/neoantigen complexes were delivered into APCs in draining LNs, elicited potent and durable neoantigen-specific T cell responses, and inhibited tumor progression

**Fig. 2:**
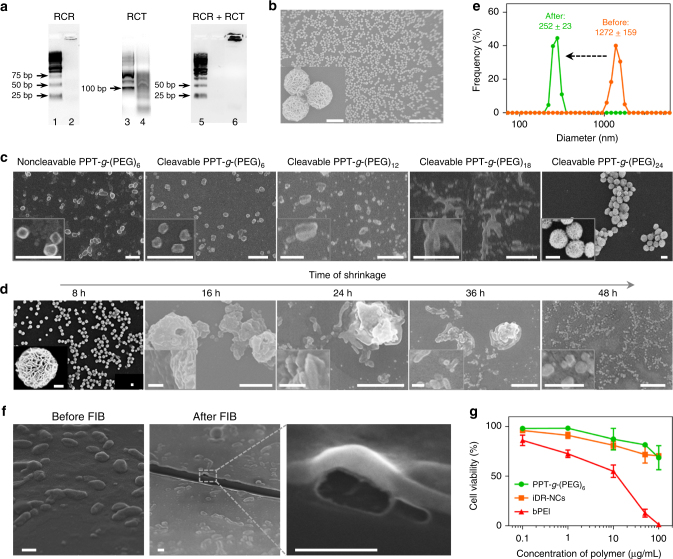
Self-assembly and characterization of iDR-NC nanoadjuvants. **a** Agarose electrophoresis images showing that long tandem CpG and shRNA were generated by separate RCR and RCT, respectively, or by combined RCR/RCT in the same solution. Lane legends: 1: 25 bp DNA ladder; 2: RCR products; 3: 100 bp DNA ladder; 4: RCT products; 5: 25 bp DNA ladder; 6: products of combined RCR/RCT. **b** SEM images showing the morphologies of CpG-shRNA MFs generated after combined RCR/RCT for 24 h. Scale bar: 50 µm; scale bar in inset: 1 µm. **c** SEM images showing the products after incubating CpG-shRNA MFs with different PPT-*g*-PEG derivatives (2.5 mg/mL) for 48 h. Scale bars: 2 µm; scale bars in inset: 1 µm. **d** SEM images showing the products after incubating CpG-shRNA MFs with 2.5 mg/mL PPT-*g*-(PEG)_6_ for a series of times. Scale bars: 2 µm; scale bars in inset: 500 nm. **e** DLS graph showing the particle sizes before (MFs) and after (iDR-NCs) shrinkage by 2.5 mg/mL PPT-*g*-(PEG)_6_. **f** SEM images showing iDR-NCs before and after FIB. FIB exposed the interior hollow structures of NCs. Scale bar: 500 nm. **g** MTS assay results showing less cytotoxicity of PPT-*g*-(PEG)_6_ and iDR-NCs than branched PEI (bPEI) in RAW264.7 macrophages

iDR-NCs were constructed in four steps: (1) Two DNA templates were designed to encode CpG 1826 (sequence: TCCATGACGTTCCTGACGTT) and *Stat3* shRNA, respectively; (2) The reaction condition was optimized to allow concurrent RCR and RCT in the same solution; (3) Combined RCR and RCT produced tandem CpG and shRNA, which were subsequently self-assembled into hybrid CpG-shRNA MFs; and (4) MFs were shrunk by positively-charged PPT-*g*-PEG copolymers to form iDR-NCs (Fig. 1). Specifically, we designed a linear phosphorylated DNA template and a DNA primer for RCR to generate tandem CpG, and a linear phosphorylated DNA template for RCT to produce tandem shRNA (Supplementary Table 1). CpG template was circularized using the DNA primer and T4 DNA ligase, and the shRNA template was circularized by CircLigase (Supplementary Fig. 1). To allow RCT and RCR in the same reaction, we optimized the reaction buffer and temperature which permitted efficient RCR (typically in RCR buffer at 30 °C) and RCT (typically in RCT buffer at 37 °C) (Supplementary Table 2). It was found that RCT, but not RCR, was susceptible to change in reaction temperature and buffer. In RCT buffer at 37 °C, RCR was still highly efficient, so this condition was chosen for combined RCR/RCT to efficiently produce nucleic acids (Fig. 2a). Altogether with magnesium pyrophosphate (Mg_2_PPi), the generated CpG and shRNA were self-assembled into CpG-shRNA MFs after 24 h reaction, as shown by SEM (Fig. 2b) and XRD analysis (Supplementary Fig. 2).

To shrink the above MFs to afford nanovaccines, PEGylated cationic polypeptide PPT-*g*-PEG was designed and synthesized, with the expectation of less cytotoxicity than conventional nucleic acid delivery polymers such as PEI. PPT-*g*-PEG was synthesized with PPTs of 45 repeating units of modified L-glutamic acids that contained 90 units of primary amines, and a series of acid-responsively-cleavable PEG grafts. The PEG was grafted onto PPTs via an acid-labile Schiff base linker. PEGylation was expected to enhance the solubility, biocompatibility, and antifouling efficacy of PPT-*g*-PEG. Upon PEG cleavage in acidic endolysosome, PEG shedding would expose cationic PPTs to further enhance endosome escape^38^. To synthesize PPT-*g*-PEG (Supplementary Fig. 3), L-glutamic acid was first modified to introduce an alkyne group using propargyl alcohol, and then triphosgene was used to transform it into N-carboxyanhydride (NCA) monomers^39,40^. PPT was synthesized by controlled ring-opening polymerization of NCA monomer via an accelerated nitrogen flow method^39^. After complete consumption of monomer as determined by ^1^H NMR, the polymer was purified by three times of precipitation into diethyl ether. Gel permeation chromatography showed monomodal molecular weight distribution with a low polydispersity index of 1.23 (Supplementary Fig. 4). Next, the pedant alkyne group was transformed into positively-charged amine via an efficient thiol-yne reaction with 2,2-dimethoxy-2-phenylacetophenone as a radical initiator under UV light. Subsequently, PEGylation was achieved by forming a Schiff base between the primary amine of PPTs and aldehyde from PEG-CHO. All structures were confirmed by ^1^H NMR (Supplementary Figs. 5–10).

The next step to construct iDR-NCs was the shrinkage of CpG-shRNA MFs using the above PPT-*g*-PEG (Fig. 1). To screen PPT-*g*-PEG for optimal shrinkage, CpG-shRNA MFs were respectively incubated with PPT-*g*-(PEG)_6_, PPT-*g*-(PEG)_12_, PPT-*g*-(PEG)_18_, and PPT-*g*-(PEG)_24_ that all had PPT backbones of 45 repeating units of modified L-glutamic acids with 6, 12, 18 and 24 copies of acid-labile PEG grafts, respectively. Note that adding one copy of PEG would reduce one positive charge in the copolymer. PPT-*g*-(PEG)_6_ with non-cleavable PEG served as a control. 48 h after incubating MFs with 2.5 mg/mL polymers in PBS, iDR-NCs were purified by centrifugation to remove free polymers. SEM revealed that PPT-*g*-(PEG)_6_ most effectively shrank the MFs to iDR-NCs, whereas PPT-*g*-PEG with 12, 18 and 24 PEG copies induced only partial shrinkage at best (Fig. 2c, Supplementary Fig. 11) likely because of insufficient positive charges and because crowded PEG grafts hindered the interaction between PPTs and MFs. Using iDR-NCs labeled with Alexa 488 via the DNA primer (Supplementary Fig. 12), flow cytometry suggested that PPT-*g*-(PEG)_6_ yielded the most efficient uptake of polymer-incubated MFs by RAW264.7 macrophages and DC2.4 cells (Supplementary Fig. 13). PPT-*g*-(PEG)_6_ was then selected for the following studies. Neither PPT-*g*-(PEG)_6_ nor PPT controls displayed NC-like structures, and PPTs without PEG failed to shrink MFs likely due to limited solubility (Supplementary Fig. 14). In a polymer concentration-dependent manner, MF shrinkage was complete with at least 2.5 mg/mL PPT-*g*-(PEG)_6_ (Supplementary Fig. 15), which was therefore used in the following studies. The process of NC formation was monitored by SEM at a series of time points after incubating MFs with PPT-*g*-(PEG)_6_. Multiple iDR-NCs gradually “bubbled” out from the outer layers of each MF at early stage, and one MF were thus shrunk to multiple iDR-NCs within a span of up to 2 days (Fig. 2d). The diameters were reduced from 1272 ± 159 nm for MFs to 252 ± 23 nm for iDR-NCs (Fig. 2e), and the zeta potential was increased from −39 mV for MFs to 1.5 mV for iDR-NCs due to the positive charge of PPT-*g*-(PEG)_6_ (Supplementary Fig. 16). iDR-NCs were further characterized by atomic force microscopy (AFM), transmission electron microscopy (TEM), and high-resolution TEM (HR-TEM) (Supplementary Fig. 17). The interior structure of iDR-NCs was studied using focused ionization beam (FIB) that cut iDR-NCs to expose the hollow interior structures of iDR-NCs, as revealed by SEM (Fig. 2f). To estimate the yield of CpG and shRNA in iDR-NCs, CpG MFs, and shRNA MFs were generated in separate RCR and RCT under the same conditions as mixed RCR/RCT, and the resulting MFs were washed to remove residual substrates and then EDTA-treated to dissolve Mg_2_PPi (Supplementary Fig. 18). By measuring absorbance at 260 nm, CpG template was estimated to be replicated for *ca*. 12 times and shRNA for *ca*. 4 times in iDR-NCs, under the assumption that all nucleic acids in MFs were shrunk into iDR-NCs. Importantly, PPT-*g*-PEG and iDR-NCs were significantly less cytotoxic than PEI in RAW264.7 macrophages, demonstrating the biocompatibility of PPT-*g*-(PEG)_6_ (Fig. 2g). Moreover, iDR-NCs showed high biostability even when incubated in 10% serum for 5 h (Supplementary Fig. 19).

### Efficient intracellular delivery of iDR-NC nanoadjuvants

**Fig. 3:**
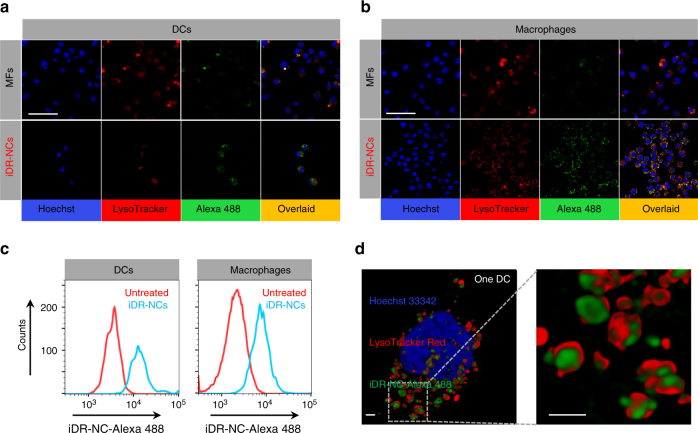
Efficient intracellular delivery of iDR-NCs into APCs. **a**, **b** Confocal microscopy images showing that, compared with the unshrunk MFs, Alexa 488-labeled iDR-NCs were more efficiently internalized into DC2.4 cells **a** and RAW264.7 macrophages **b**. Scale bar: 50 µm. Hoechst: Hoechst 33342. LysoTracker: LysoTracker Red DND-99. Efficiently internalized iDR-NCs were co-localized with endolysosome after 3-h incubation. **c** Flow cytometry graphs showing efficient internalization of Alexa 488-labeled iDR-NCs by DC2.4 cells and RAW264.7 macrophages after 3 h. Red: untreated cells; blue: cells treated with iDR-NCs. **d** 3D-deconvolved confocal microscopy images showing one DC2.4 cell that internalized iDR-NCs into its endolysosome and cytosol after 3-h incubation. Scale bar: 1 µm. (iDR-NC concentration: 500 nM CpG equivalents)

As TLR9 (receptor for CpG) resides in the endolysosomes of APCs and *Stat3* mRNA is in the cytosol, efficient intracellular delivery of iDR-NCs is essential for potent immunomodulation. After Alexa 488-labeled iDR-NCs were incubated with RAW264.7 macrophages and DC2.4 cells for 3 h, efficient intracellular delivery of iDR-NCs was observed by confocal microscopy (Fig. 3a, b) and flow cytometry (Fig. 3c). Specifically, confocal microscopy revealed massive colocalization of internalized iDR-NCs with the endolysosome stained by LysoTracker Red, and owing to shrinkage, the delivery efficiency of iDR-NCs dramatically outperformed the parent MFs (Fig. 3a, b). Confocal microscopy with 3D deconvolution clearly revealed iDR-NCs in the endolysosome of DC2.4 cells, which would allow binding of CpG with TLR9 for immunostimulation (Fig. 3d; Supplementary Fig. 20; Supplementary Movie 1). As shRNA and CpG were co-incorporated into iDR-NCs, these results suggest efficient intracellular delivery of both CpG and shRNA. We hypothesize that iDR-NCs would be dissociated in the acidic endolysosome to liberate CpG, and the acid-labile linker in PPT-*g*-(PEG)_6_ would break in acidic endolysosome so that the exposed cationic PPTs in iDR-NCs would facilitate the endosomal escape of shRNA via proton sponge effect. Overall, these results provide the basis for intracellular immunomodulation by iDR-NC nanovaccines.

### iDR-NC/antigen co-delivered adjuvants and antigens to APCs

Co-delivery of adjuvants and antigens into APCs is key for optimal antigen presentation. To study this, we used Alexa 555-labeled iDR-NCs and model antigen CSIINFEKL, an epitope of chicken ovalbumin (OVA) with a cysteine appended on the N-terminal. To monitor the behaviors by fluorescence, CSIINFEKL was modified with FITC on lysine that was reported not to affect its binding with major histocompatibility complex I (MHC I)^41^. CSIINFEKL was loaded into iDR-NCs through physical complexation driven by hydrophobic interactions between CSIINFEKL and PTTs. Specifically, iDR-NCs (from 200 µL RCT/RCR products) were co-incubated with CSIINFEK_(FITC)_L (100 µM) at room temperature for 5 h, followed by washing with PBS and centrifugation for three times to remove free antigen. As determined by the FITC fluorescence of unloaded antigens, the amount of CSIINFEKL loaded into the resulting iDR-NC/CSIINFEK_(FITC)_L complexes (SEM images in Supplementary Fig. 21) was 2.1 molar equivalents of CpG (CpG: CSIINFEKL = 1: 2.1).

**Fig. 4:**
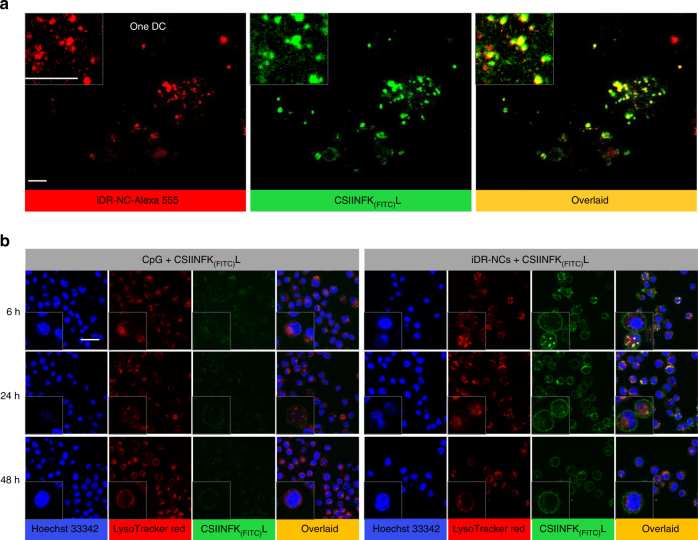
iDR-NCs induced efficient and sustained antigen presentation. **a** Instant SIM super-resolution images showing one DC with apparent colocalization between iDR-NC-Alexa 555 and CSIINFEK_(FITC)_L. Scale bar: 1 µm. **b** Confocal microscopy images showing that, relative to CpG, iDR-NCs enhanced the internalization and antigen presentation of CSIINFEK_(FITC)_L in BMDCs. BMDCs were incubated with iDR-NC/CSIINFEK_(FITC)_L complexes or CpG + CSIINFEK_(FITC)_L for 14 h (except for 6-h point), washed, and further incubated for a total of 24 h and 48 h before observation. (CpG: 100 nM equivalents, CSIINFEKL: 210 nM.) Insets show zoom-in views of single cells. Scale bar: 20 µm

Intracellular co-delivery in DCs was then investigated by super resolution fluorescence imaging using a home-built instant structured illumination microscope (instant SIM) that enables the high-speed acquisition of super resolution images with relatively little photobleaching (which was critical in imaging the easily photobleached FITC)^42^. Specifically, DC2.4 cells were incubated with iDR-NC-Alexa 555/CSIINFEK_(FITC)_L complexes for 6 h, then washed before super resolution imaging. Instant SIM revealed a high degree of colocalization of iDR-NCs with CSIINFEK_(FITC)_L in DCs (Fig. 4a), suggesting that iDR-NCs is co-delivered with antigen and providing the basis for efficient antigen presentation. To examine antigen presentation, bone marrow derived dendritic cells (BMDCs) were incubated with iDR-NCs/CSIINFEK_(FITC)_L complexes for a total of 6, 24, and 48 h before confocal microscopy observation. For 24-h and 48-h points, cells were washed at 14 h after adding iDR-NCs/CSIINFEK_(FITC)_L, followed by further incubation to investigate the sustainability of antigen presentation. Compared to free CpG control, iDR-NCs not only enhanced the uptake of CSIINFEK_(FITC)_L but also resulted in sustained presence of CSIINFEK_(FITC)_L on BMDC surfaces, likely resulting from sustained antigen presentation of CSIINFEK_(FITC)_L (Fig. 4b). Note that, at early stage, both free CSIINFEK_(FITC)_L and CSIINFEK_(FITC)_L loaded in iDR-NCs were efficiently internalized into BMDCs and presented on BMDC surfaces, likely due to efficient uptake of particulate CSIINFEK_(FITC)_L (formed by hydrophobic interactions) as well as strong epitope binding of CSIINFEK_(FITC)_L with MHC I on BMDC surfaces.

### Synergistic in vitro immunoactivation by CpG/shRNA iDR-NCs

**Fig. 5:**
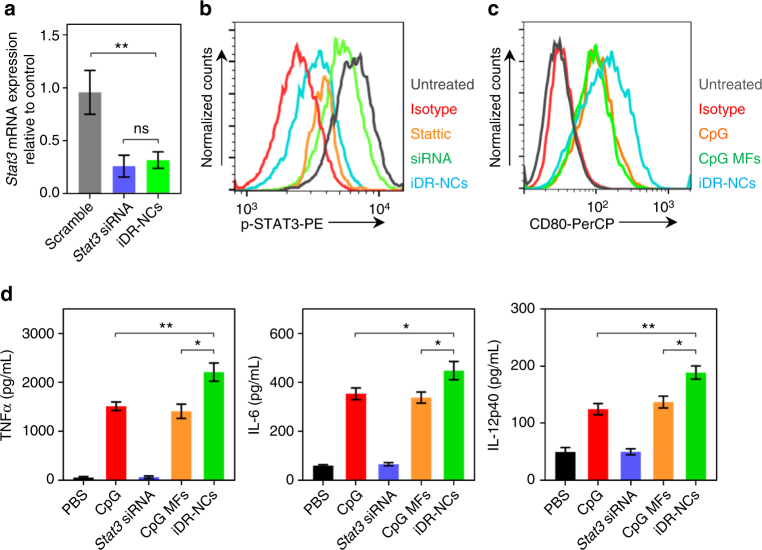
Synergistic in vitro immunostimulation of iDR-NC nanoadjuvants. **a** Real-time quantitative RT-PCR results showing *Stat3* silencing in DC2.4 cells treated with iDR-NCs for 48 h. *Stat3* siRNA and scramble served as controls. **b** Flow cytometry results showing that p-STAT3 expression was down-regulated in DC2.4 cells treated with iDR-NCs for 48 h. STAT3 inhibitor (Sttatic) and Lipofectamine2000-transfected *Stat3* siRNA were used as controls. *Stat3* shRNA: 100 nM equivalents in **a**, **b**. **c** Flow cytometry results showing that iDR-NCs (100 nM CpG equivalents) elevated the expression of costimulatory factor CD80 in DC2.4 cells after 24 h. **d** ELISA results suggest iDR-NCs (200 nM CpG equivalents) induced DC2.4 cells to secrete significantly more TNFα, IL-6, and IL-12p40 than CpG or CpG MFs, after 24 h. *Stat3* siRNA alone was used as a control. (ns: non-significant; *p < 0.05; **p < 0.01; *n* = 3; one-way ANOVA with Bonferroni post-hoc test). Data represented as mean ± s.e.m.

We studied *Stat3* silencing in DC2.4 cells that were treated with iDR-NCs for 48 h. Real-time quantitative RT-PCR of mRNA from the treated cells demonstrated the specific silencing of *Stat3*, compared with a glyceraldehyde 3-phosphate dehydrogenase (GAPDH) (Fig. 5a). Treated DCs were further analyzed for STAT3 phosphorylation at tyrosine 705 (Y705), a prerequisite of STAT3 activation via dimerization, translocation into nucleus, DNA binding, and terminal differentiation and growth arrest in monocytes in response to cytokine stimulation. Specifically, the as-treated DC2.4 cells were permeabilized and intracellularly stained using an antibody against phosphorylated STAT3 (p-STAT3). Lipofectamine-transfected *Stat3* siRNA and Stattic, a STAT3 inhibitor, served as controls. Flow cytometry suggested that iDR-NCs downregulated p-STAT3 (Fig. 5b), at higher efficiency than siRNA, likely due to efficient delivery and biocompatibility of iDR-NCs relative to Lipofectamine.

iDR-NCs were then investigated for immunostimulation in RAW264.7 macrophages and DC2.4 cells. Cells were treated with iDR-NCs for 24 h and analyzed for cell-surface expression of costimulatory factor CD80 and the secretion of proinflammatory factors interleukin-6 (IL-6), IL-12p40, and tumor necrosis factor α (TNFα). In DC2.4 cells, iDR-NCs elevated CD80 expression, at a level higher than phosphorothioate-stabilized CpG or parental CpG MFs, which is attributed to efficient intracellular iDR-NC delivery as well as synergistic *Stat3* silencing and CpG immunostimulation (Fig. 5c). Consistently, ELISA showed that, compared with CpG or CpG MFs, treatment with iDR-NCs significantly enhanced the secretion of IL-6, IL-12p40, and TNFα in DC2.4 cells (Fig. 5d). As expected, *Stat3* siRNA alone was not immunostimulatory. Similarly, iDR-NCs activated RAW264.7 macrophages to elevate CD80 expression and proinflammatory factor secretion (Supplementary Fig. 22). Altogether, these results suggest the potent and synergistic immunostimulatory effects of iDR-NCs.

### Delivery of iDR-NCs to LNs and LN APCs in mice

**Fig. 6:**
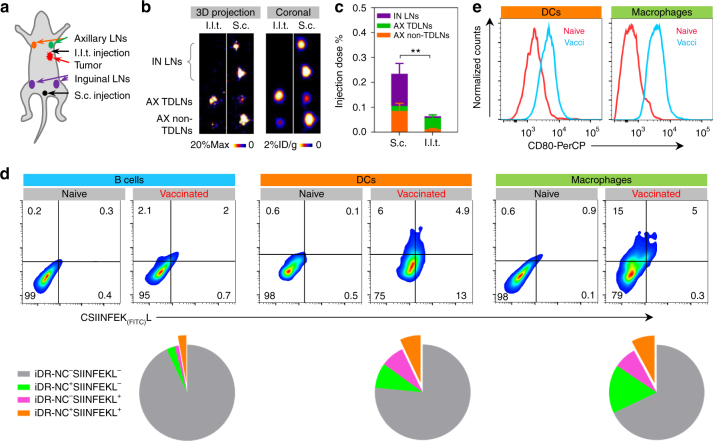
Efficient delivery of iDR-NCs to LN APCs in vivo. **a** Schematic illustration of the LN delivery study of iDR-NCs. **b**, **c** Representative 3D projection and coronal PET views **b** and ID% **c** of ^64^Cu-labeled iDR-NCs in LNs one day after i.l.t. or s.c. injection. **d** Upper: representative flow cytometry plots showing the signals of Alexa 555-labeled iDR-NCs and CSIINFEK_(FITC)_L in LN-residing B220^+^ B cells, CD11c^+^ DCs, and F4/80^+^ macrophages one day after s.c. injection at the tail base of C57BL/6 mice. Lower: pie charts showing the quantified flow cytometry results in vaccinated mice. (*n* = 4 in **a**–**d**; **p < 0.01; one-way ANOVA with Bonferroni post-hoc test). Data represent mean ± s.e.m. **e** Flow cytometry results showing elevated CD80 expression in LN DCs and macrophages one day after s.c. vaccination (vacci.) of iDR-NCs in C57BL/6 mice. Age-matched naive mice were used as controls in **b**, **c**

We then studied the in vivo delivery and immunostimulation of iDR-NCs in immunocompetent C57BL/6 mice. Locally administered nanovaccines exploit the physiological characteristics of lymphatic drainage to transport nanovaccines via lymphatics to LNs, where various lymphocytes reside and immune responses are orchestrated. The sizes of iDR-NCs fell within the nanoparticle size range that allow minimal systemic dissemination and efficient lymphatic drainage for LN accumulation^31^, which motivated us to study LN delivery of these nanoadjuvants. To this end, iDR-NCs were prepared using NOTA-modified PPT-*g*-(PEG)_6_ and then radiolabeled with ^64^Cu through NOTA chelator. ^64^Cu-labeled iDR-NCs were injected subcutaneously (s.c.) at the tail base or ipsilateral to tumor (i.l.t.), respectively, to C57BL/6 mice bearing MC38 syngeneic colon adenocarcinoma tumor (Fig. 6a). I.l.t. injection features proximity with tumor-derived antigens in its tumor-draining LNs (TDLNs). At 1 day post injection, draining inguinal (IN) LNs and axillary (AX) LNs were resected to quantify the radioactivity by γ counting, as well as positron emission tomography (PET). S.c. injection delivered a total of 3-fold more iDR-NCs than i.l.t. injection into IN and AX LNs (Fig. 6b, c). Specifically, the percent injected dose (%ID) of s.c.-injected iDR-NCs was 0.13% in IN LNs and 0.11% in AX LNs. In contrast, only 0.046% of i.l.t.-injected iDR-NCs were delivered to AX TDLNs and a total of <0.02% of iDR-NCs were in IN and AX non-TDLNs (Fig. 6c). Despite the potential of TDLNs to harbor tumor antigens, we chose s.c. injection because of its relatively efficient LN delivery and that exogenous MC38-specific neoantigen was used together with iDR-NCs for antigen-specific immunomodulation in this study.

As mentioned, potent antigen presentation on APC surfaces and effective immunomodulation demand co-delivery of adjuvants and antigens into APCs. We thus studied co-delivery of Alexa 555-labeled iDR-NC/CSIINFEK_(FITC)_L complexes after s.c. injection at the tail base of C57BL/6 mice. At 1 day post injection, draining IN LNs were resected for flow cytometric analysis of Alexa 555 and FITC signals in B220^+^ B cells, F4/80^+^ macrophages, and CD11c^+^ DCs, which are major populations of APCs that can present antigens to naive T cells. 18.2% DCs and 25.4% macrophages were Alexa 555^+^FITC^+^, demonstrating efficient co-delivery of iDR-NCs with peptide antigen to LN APCs in vivo (Fig. 6d). In comparison, Alexa 555^+^FITC^+^ B cells were <1%. The efficient in vivo uptake of NCs and antigens was attributed to shrunk sizes of iDR-NC/CSIINFEK_(FITC)_L complexes. Further, at 1 day after iDR-NC immunization, CD80 expression was elevated in LN DCs and macrophages, indicating in vivo APC activation (Fig. 6e). The efficient co-delivery and immunostimulation of iDR-NCs in vivo are expected to elicit potent T cell responses.

### iDR-NC/neoantigen potentiated T cell responses with memory

**Fig. 7:**
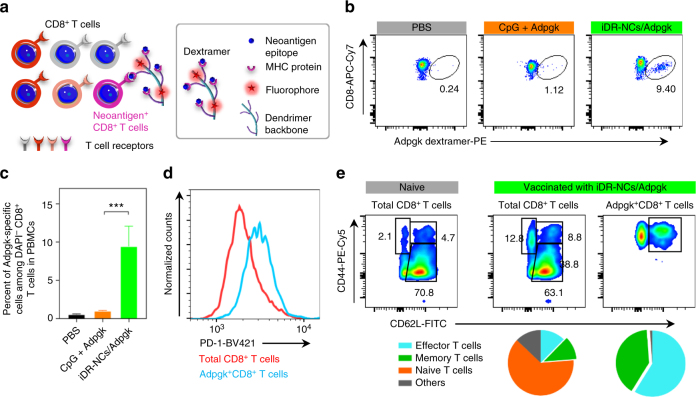
iDR-NC/neoantigen elicited potent and durable neoantigen-specific T cell responses. C57BL/6 mice were s.c. immunized with CpG/shRNA^*Stat3*^-iDR-NCs/Adpgk (2 nmol CpG equivalents, 17 μg Adpgk) on day 0 and day 14. **a** Schematic illustration of dextramer staining to analyze antigen-specific CD8^+^ T cells. **b** Representative flow cytometry and **c** quantification of ASMTNMELM-specific CD8^+^ T cells among live (DAPI^−^) CD8^+^ cells in PBMCs, as stained using a PE-conjugated ASMTNMELM-H-2D^b^ dextramer on day21. **d** Flow cytometry showing upregulated PD-1 expression on PBMC Adpgk^+^CD8^+^ T cells, relative to total CD8^+^ T cells, in mice vaccinated with iDR-NC/Adpgk on day21. **e** Upper: representative flow cytometry plots showing CD8^+^ T cell effector/effector memory/central memory phenotypes in PBMCs of naive mice and mice vaccinated with iDR-NC/Adpgk on day49; lower: quantification of the fractions of CD8 T cell populations in mice vaccinated with iDR-NC/Adpgk. iDR-NC/Adpgk induced substantial central memory (CD62L^high^CD44^+^) CD8^+^ T cells, especially memory Adpgk^+^CD8^+^ T cells compared with age-matched naive mice. (***p < 0.001; *n* = 4; one-way ANOVA with Bonferroni post-hoc test). Data represent mean ± s.e.m.

T cell responses against neoantigen-presenting tumor cells play a central role in cancer immunotherapy. To study iDR-NC/antigen for neoantigen-specific cancer immunotherapy, we attempted to analyze T cell responses after immunizing C57BL/6 mice with iDR-NC/neoantigen complexes. Neoantigens were generated from somatic tumor mutation and were identified by exome sequencing and mass spectrometry. We used neoantigen Adpgk generated from mutation (ASMTNRELM → ASMTNMELM) in MC38 tumor^37^. Again, iDR-NC/neoantigen complexes (SEM images in Supplementary Fig. 21b) were prepared by co-incubating iDR-NCs with hydrophobic Adpgk to allow physical complexation of Adpgk with the PPT moieties via hydrophobic interactions. By quantifying the unloaded Adpgk using UV absorbance (280 nm), the amount of Adpgk loaded into iDR-NCs was 2.7 molar equivalents of CpG (CpG: Adpgk = 1: 2.7). Syngeneic C57BL/6 mice were immunized with CpG/shRNA^*Stat3*^-iDR-NC/Adpgk complexes (2 nmol CpG equivalents, 17 μg Adpgk) on day 0 for priming and day 14 for boosting. On day 21, peripheral blood was collected, red blood cells lysed, and CD8^+^ T cells in peripheral blood mononuclear cells (PBMCs) were stained using a PE-conjugated H-2D^b^-ASMTNMELM dextramer. This dextramer is a molecular complex that mimics MHC I^−^antigen epitope complexes presented on cancer cells, such that it can stain T cells that are able to recognize Adpgk-presenting cancer cells (Fig. 7a. Supplementary Fig. 23). Control CpG + Adpgk elicited only 1.1% ASMTNMELM-specific CD8^+^ T cells among all live (DAPI^−^) CD8^+^ cells. In contrast, iDR-NC/Adpgk induced 9.5% ASMTNMELM-specific CD8^+^ T cells (Fig. 7b, c), an 8-fold increase relative to CpG+Adpgk. Moreover, these ASMTNMELM-specific CD8^+^ T cells in nanovaccine-immunized mice upregulated the expression of programmed death receptor 1 (PD-1) (Fig. 7d), an immune checkpoint whose expression on T cells, especially antigen-specific CD8^+^ T cells, can be upregulated by immunostimulation^43^. Thus, the upregulated PD-1 expression on ASMTNMELM-specific CD8^+^ T cells further implied potent immunostimulation of iDR-NC/Adpgk. Remarkably, on day 49, mice immunized with iDR-NC/Adpgk showed large fractions of central memory CD8^+^ T cells (CD8^+^CD44^+^CD62L^high^), especially Adpgk-specific memory CD8^+^ T cells (ASMTNMELM^+^CD8^+^CD44^+^ CD62L^high^) (Fig. 7e), indicating durable T cell responses. Again, the potent and durable immunity was attributed to the potent synergistic immunostimulation of iDR-NCs as well as the co-delivery of Adpgk with iDR-NCs via iDR-NCs/Adpgk complexes.

### iDR-NC/neoantigen nanovaccines for cancer immunotherapy

**Fig. 8:**
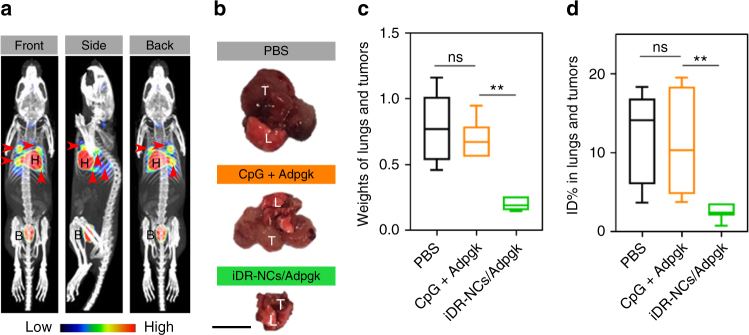
iDR-NC/neoantigen as potent nanovaccines for neoantigen-specific tumor immunotherapy. 1 × 10^5^ MC38 cells were *i*.*v*. injected into C57BL/6 mice, and mice were treated by s.c. injection of CpG/shRNA*Stat3*/neoantigen iDR-NCs (2 nmol CpG equivalents, 17 μg Adpgk) at the tail base on days 10, 16, and 22. **a** Representative PET-CT images showing the MC38 tumor deposited in lungs on day 40. Mice were randomly picked from the group of PBS-treated mice for PET-CT. Tumor, as well as heart and bladder were illuminated by FDG. Arrow heads mark tumor nodules. *H* heart, *B* bladder. **b** Representative photographs (scale bar: 1 cm.), **c** weights, and **d** %ID of FDG of lungs and tumors from as-treated mice on day 40 post tumor inoculation. *T* tumor, *L* lung. (**p < 0.01; ***p < 0.001; ns: not significant; *n* = 6; one-way ANOVA with Bonferroni post-hoc test). Data represent mean ± s.e.m.

Motivated by the potent and durable T cell responses induced by iDR-NC/neoantigen complexes, we then studied tumor immunotherapy using the above nanovaccines. In addition to durable immunity, another characteristic of immunotherapy is systemic efficacy. We studied neoantigen-specific immunotherapy of MC38 tumor in syngeneic C57BL/6 mice. MC38 cells (1 × 10^5^) were *i*.*v*. injected into C57BL/6 mice. On day10, mice were treated with iDR-NC/Adpgk complexes (2 nmol CpG equivalents, 17 μg Adpgk), followed by boosting on day 16 and day 22. At the end of the study (day 40), the metabolic activity of metastatic-like tumors in lungs was determined using ^18^F-fludeoxyglucose (FDG), a metabolic radiotracer for tumor diagnosis. At 1 h post FDG injection, tumor burden in lung from randomly picked PBS-treated mice were clearly illuminated in PET-CT and showed apparently higher FDG signal intensities than background muscle (Fig. 8a). Hearts and bladders also showed intense signals due to high metabolic activity and renal clearance, respectively. Mice were euthanized and organs of interest (lung and tumor together, heart, liver, and muscle) were resected, weighted, and radioactivity measured by γ counting. Tumor and lung together showed 21-fold higher uptake density (reflected by %ID per g) of FDG than background muscle (Supplementary Fig. 24). The total weight of lung and tumor from mice treated with iDR-NC/Adpgk was reduced by 5 times compared with that of free vaccines (Fig. 8b, c). Consistently, the FDG radioactivity of lung and tumor from mice treated with iDR-NC/Adpgk were significantly less than that of control groups (Fig. 8d). Altogether, iDR-NC/Adpgk potently and systemically inhibited tumor progression in neoantigen-specific personalized tumor immunotherapy.

## Discussion

Nanovaccines are an important class of nanomedicines that hold substantial potential for disease treatment including cancer immunotherapy. Nucleic acid nanovaccines are particularly attractive because (1) the structural programmability of nucleic acids enables precise engineering of the nanostructure morphology and pinpoint loading of theranostic agents, and (2) nucleic acids have intrinsic immunomodulatory functionalities (e.g., immunostimulatory CpG and poly(I:C)). These characteristics make nucleic acid nanovaccines especially appealing for combination cancer immunotherapy, for which nanovaccines that can co-deliver DNA, RNA, and peptide therapeutics are of significant interest. For example, immunomodulatory DNA and RNA adjuvants can exploit independent signaling pathways to synergistically elicit antitumor immunity, and combination of these nucleic acid adjuvants with peptide antigens can elicit potent and durable T cell responses, which is critical for efficacious cancer immunotherapy.

In this study, we first developed nucleic acid nanomaterials, termed iDR-NCs, that were self-assembled from both DNA and RNA in a two-in-one reaction system; we then constructed immunotherapeutic nanovaccines by loading tumor neoantigens into iDR-NCs. Specifically, we optimized RCR/RCT reactions in the same solution to concurrently generate tandem DNA and RNA, which were further self-assembled into intertwining DNA-RNA MFs. While separate RCR or RCT have been previously employed to construct DNA or RNA nanostructures^5,16^, iDR-NCs represent the first hybrid DNA-RNA nanostructures that were generated via combined RCR and RCT in the same reaction system. The combined RCR and RCT essentially incorporated both DNA and RNA therapeutics into the single nanomedicine. Specifically, we engineered MFs to be assembled of synergistic CpG and *Stat3*-silencing shRNA, such that TLR9 and STAT3 signaling pathways were synergistically leveraged to elicit potent immunostimulation in APCs for cancer immunotherapy. To improve delivery to LNs and LN APCs while maintaining the biosafety of nanovaccines, we synthesized a biocompatible PPT-*g*-PEG copolymer to shrink MFs into NCs. Although polyethylenimine (PEI) may also shrink this type of microstructures, the cytotoxicity of highly cationic PEI raises serious safety concerns^16^. To address this issue, we synthesized biocompatible PEG-grafted polypeptides (PPT-*g*-PEG) copolymers to shrink MFs. Further, given the hydrophobicity of PPTs in this copolymer, we physically loaded tumor-specific neoantigens into iDR-NCs via hydrophobic interactions between neoantigens and PPTs. Most neoantigens are hydrophobic, thus loading neoantigens into iDR-NCs by hydrophobic interactions is expected to be widely applicable to most neoantigens. Furthermore, hydrophilic neoantigens could be loaded into iDR-NCs by chemically conjugating neoantigens with PPT-*g*-PEG. Moreover, this multifunctional PPT-*g*-PEG is expected to shed PEG in the acidic endolysosome of APCs to enhance the proton sponge effect of cationic PPTs and then facilitate cytosolic delivery of shRNA and peptide neoantigens. Using quantitative PET imaging, super-resolution fluorescence imaging and flow cytometry, we demonstrated that iDR-NC/neoantigen nanovaccines were efficiently delivered to LNs and LN APCs. We further showed that CpG/shRNA^*Stat3*^-iDR-NCs synergistically activated APCs in vitro. Although the synergistic efficacy between CpG and shRNA^*Stat3*^ for in vivo T cell responses or tumor immunotherapy has yet to be investigated, the potent T cell responses and tumor therapeutic efficacy of iDR-NC/Adpgk are presumably attributed, at least in part, to this synergistic APC activation.

Potent and durable neoantigen-specific antitumor T cell responses were elicited by iDR-NC/Adpgk nanovaccines. Neoantigens and neoantigen-specific CTLs have been preclinically and clinically shown to be pivotal in tumor immunotherapy^35,36,44–46^. However, natural neoantigen-specific CTLs are extremely rare (e.g., < 0.001% in colorectal cancer^47,48^), likely due to low clonal neoantigen burden, inefficient antigen processing and cross presentation, as well as immunosuppression. Delivering exogenous neoantigens can enhance the frequencies of neoantigen-specific CTLs to improve cancer immunotherapy. Therefore, nanovaccines co-delivering synergistic adjuvants and neoantigens are promising to potentiate neoantigen-specific immunity for tumor immunotherapy^29,33^. In this study, iDR-NC/Adpgk nanovaccines increased the frequency of Adpgk-specific CD8^+^ CTLs, which contributed to significant inhibition of tumor progression. Worth noting, the potent immunotherapeutic efficacy of these nanovaccines in metastatic-like tumor models is particularly significant, given the fact that cancer metastasis is a primary cause of death of cancer patients and the ability to effectively treat metastatic tumors is thus highly desirable.

Combination immunotherapy has been extensively explored in numerous clinical trials. In the present study, even though no tumors were regressed by iDR-NC/Adpgk, we believe that combination of these nanovaccines with other therapeutics, such as checkpoint inhibitors, multi-epitope antigens, antitumor cytokines, as well as chemotherapeutics, have the potential to further improve therapeutic efficacy and increase regression rates by exploiting synergistic immunostimulating signaling pathways. Intriguingly, upon immunization with iDR-NC/Adpgk, the expression of immune checkpoint PD-1 was upregulated on CD8^+^ T cells. While this PD-1 upregulation suggests T cell exhaustion, it also implies that combination of PD-1 inhibitors with iDR-NC/Adpgk could potentially enhance the therapeutic efficacy. Furthermore, iDR-NCs are expected to deliver multiple tumor antigen to LNs for a broader spectrum of T cell responses, which could drive broader tumor cell killing and therefore better therapeutic efficacy.

Taken together, iDR-NCs represent a general technology to construct hybrid DNA-RNA nanostructures that can be further loaded with hydrophobic peptide therapeutics. Specifically, in this study, CpG and *Stat3* shRNA were used as building blocks, with PPT-*g*-PEG copolymers as shrinkage materials. The resulting iDR-NCs were further loaded with tumor neoantigens and served as potent synergistic nanovaccines for personalized combination cancer immunotherapy.

## Methods

### DNA synthesis

DNA was synthesized using reagents from Glen Research (Sterling, VA) and Chemgenes (Wilmington, MA), on an Applied Biosystems ABI 392 DNA synthesizer. DNA was deprotected according to manufacturer’s instructions, followed by purification on a RP-HPLC (Dionex Ultimate 3000, ThermoFisher Scientific, Waltham, MA). Dye-labeled DNAs were purchased from IDT (Coralville, IA).

### Synthesis of PPT-*g*-PEG

*Synthesis of γ-propargyl-L-glutamate hydrochloride*: L-glutamic acid (6.0 g, 40.8 mmol) was suspended in 200 mL propargyl alcohol. 14.3 mL of chlorotrimethylsilane was added into the suspension dropwise over 2 h at room temperature, followed by further stirring for 2 days. The reaction mixture was purified by precipitation into diethyl ether, and further purified by three-times precipitation from methanol (50 mL) to diethyl ether (0.5 L). The product was filtrated and dried (4.71 g, yield: 52 %). ^1^H NMR (300 MHz, CD_3_OD, ppm): δ 4.73 (d, 2 H), 4.05 (t, *J* = 6.7 Hz, 1 H), 2.95 (t, *J* = 2.5 Hz, 1 H), 2.65 (m, 2 H), 2.39–2.11 (m, 2 H).

*Synthesis of γ-propargyl-L-glutamate N-carboxyanhydride*: γ-propargyl-L-glutamate hydrochloride (2.0 g, 9.0 mmol) and triphosgene (2.7 g, 3.9 mmol) were suspended in 100 mL ethyl acetate, and were then stirred under reflux for 3 h until the reaction mixture became clear. The crude product was extracted with 100 mL cold nanopure water, 100 mL cold saturated sodium bicarbonate solution, and 100 mL cold saturated sodium chloride solution. The organic layer was dried over MgSO_4_, concentrated to remove ethyl acetate. Yellow viscous oil was obtained after twice precipitation from ethyl acetate into hexane and dried (1.23 g, yield: 65%). ^1^H NMR (300 MHz, CDCl_3_, ppm): δ 6.76 (s, 1 H), 4.71 (d, *J* = 2.5 Hz, 2 H), 4.44 (ddd, *J* = 6.7, 5.7, 0.9 Hz, 1 H), 2.61 (t, *J* = 7.0 Hz, 2 H), 2.52 (t, *J* = 2.5 Hz, 1 H), 2.39 − 2.12 (m, 2 H).

*Synthesis of PPT*: The PPT was synthesized using ring-opening polymerization. A 25-mL dry Schlenk flask was charged with 3-methoxypropylamine (5.7 mg, 1 equivalent (equiv.)), and PLG NCA (610 mg, 45 equiv.) in anhydrous DMF (15 mL). Continuous nitrogen flow was used to promote polymerization at room temperature. ^1^H NMR was used to monitor the polymerization and <99% monomer was consumed in 20 h. The polymer was purified by three times’ precipitation from DMF into diethyl ether under vigorous stirring and washed with diethyl ether to afford a white powder after drying in vacuum at room temperature (430 mg, yield: 70%). GPC results (M_n_ 15145 Da, M_w_ 18691 Da, PDI: 1.23, using poly(methyl methacrylate)s as calibration standards). ^1^H NMR (300 MHz, DMSO-d_6_) δ 8.14 (s, 33 H), 4.67 (d, *J* = 1.9 Hz, 71 H), 4.24 (s, 34 H), 3.51 (m, 35 H), 3.29 (t, *J* = 6.7 Hz, 3 H), 3.21 (s, 3 H), 3.10 (m, 2 H), 2.47 − 2.27 (m, 57 H), 1.78 (m, 67 H).

*Synthesis of cationic PPT cysteamine hydrochloride*: PPT_45_ **3** (250 mg, 1 eq), DMPA (212 mg, 25 equiv.), and cysteamine hydrochloride (3385 mg, 900 equiv.) were added into 10 mL anhydrous DMF in a 50-mL flask. The reaction mixture was then bubbled by nitrogen flow for 10 min, and placed under UV light with a wavelength of 365 nm for 2.5 h. The mixture was transferred into a presoaked dialysis membrane tubing (MWCO 6–8 kDa), dialyzed against nanopure water for another 2 days, and then lyophilized to get the final product (568 mg, yield: 86%). ^1^H NMR (300 MHz, DMSO-d_6_) δ 8.61–7.48 (br, 167 H) 4.40 (s, 90 H), 3.55 (s, 40 H), 3.2 (m, 42 H), 2.99 (s, 105 H), 2.81 (br, 153 H), 2.48–2.12 (m, 70 H), 2.02–1.56 (m, 69 H).

*Synthesis of PEG-CHO*: Methoxyl polyethylene glycol (PEG_45_, 10.0 g, 1 equiv.), 4-formylbenzoic acid (1.5 g, 2 equiv.), N-(3-dimethylaminopropyl)-N′-ethylcarbodiimide hydrochloride (EDC, 1.9 g, 2 equiv.), and 4-(dimethylamino)pyridine (305 mg, 0.5 equiv.) were mixed in 100 mL dichloromethane in a 250 mL flask. The reaction mixture was stirred for 24 h at room temperature, and then was washed with saturated sodium bicarbonate aqueous solution (1 × 100 mL), and saturated brine (3 × 100 mL). The organic layer was separated and dried over anhydrous Na_2_SO_4_. The organic solvent was then concentrated by rotatory evaporator to obtain final product (9.3 g, yield: 87%). ^1^H NMR (300 MHz, CDCl_3_) δ 10.12 (s, 1 H), 8.25–8.20 (m, 2 H), 8.00–7.94 (m, 2 H), 4.55–4.50 (m, 2 H), 3.66 (s, 138 H), 3.39 (s, 3 H).

*Synthesis of PPT-g-PEG*: Cationic PPTs (100.0 mg, 1 equiv.), PEG-CHO (60.3 mg, 6 equiv.), and trimethylamine (137.0 mg, 270 equiv.) were added into 5 mL anhydrous DMSO in a 20-mL vial. The reaction mixture was stirred for 24 h and monitored by ^1^H NMR to confirm the complete consumption of PEG-CHO. The mixture was transferred into a presoaked dialysis membrane tubing (MWCO 6–8 kDa), dialyzed against nanopure water (pH 7.4) for another 5 h, changing water every hour, and then lyophilized to get the final product (142 mg, yield: 83%). ^1^H NMR (300 MHz, DMSO-*d*_6_) δ 8.46 (s, 1 H), 8.04 (d, *J* = 8.1 Hz, 2 H), 7.90 (d, *J* = 8.1 Hz, 2 H) 8.65–7.65 (m), 4.45 (s, 152 H), 3.94–3.56 (m), 3.52 (s, 167 H), 3.25 (s, 3 H), 2.94–2.60 (m), 2.28 (q, *J* = 7.2 Hz, 3 H), 2.13 (s).

### Synthesis of PPT-g-PEG-NOTA

PPT-*g*-PEG (2.1 mg, 1 equiv.), NHS-NOTA (0.22 mg, 10 equiv.), and N,N-diisopropylethylamine (0.84 mg, 100 equiv.) were added into 1 mL anhydrous DMSO in a 20-mL vial. The mixture was stirred for overnight and unreacted NOTA was removed using PD-10 columns (GE Healthcare Life Sciences).

### Self-assembly of iDR-NCs and iDR-NC/antigen complexes

Hybrid DNA-RNA MFs were first synthesized using mixed RCR/RCT. For RCR, linear 5′-phosphorylated CpG DNA templates (0.6 µM) were circularized using ligation helper DNA (1.2 µM) and T4 DNA ligase (10 U/µL; New England Biolabs, Ipswich, MA). This helper DNA also served as the primer for RCR. RCR reaction system contained circular template (0.3 µM), Φ29 DNA polymerase (2 U/µL), 2 mM dNTP, and 1× BSA in buffer solution (40 mM Tris–HCl, 6 mM MgCl_2_, 2 mM spermidine, 1 mM dithiothreitol) (New England Biolabs, Ipswich, MA). For RCT, STAT3 linear template was circularized using CircLigase according to manufacturer’s instructions (Epicentre, Charlotte, NC). Circular template was directly used for RCT. A typical RCT system contains template (2.5 µM), T7 RNA polymerase, 2 mM NTP, 1× BSA, and 1× RCT reaction buffer (40 mM Tris–HCl, 6 mM MgCl_2_, 2 mM spermidine, 1 mM dithiothreitol) (New England Biolabs, Ipswich, MA). RCR was carried out at 37 ^o^C for a specified time. Reaction was terminated by deactivation at 90 °C for 10 min. The products were washed twice using PBS and centrifugation (14,000 rpm, 10 min), and resuspended in PBS before use. If applicable, Alexa 488 or Alexa 555 was labeled on the 5′ end of primers used for RCR; alternatively, fluorescein-modified UTP (Enzo Life Sciences, Farmingdale, NY) were added into the above reaction system for fluorophore labeling in MFs. Then, DNA-RNA MFs were shrunk into NCs by incubating the MFs with copolymers. In a typical experiment, 2.5 mg/mL copolymer in PBS was mixed with MFs on a vortex for 48 h. At the end of shrinkage, the mixture was centrifuged (14,000 rpm, 10 min) to remove supernatant, followed by washing twice using PBS.

iDR-NC/antigen complexes were prepared by physical complexation of hydrophobic antigens with PPT moieties in iDR-NCs. After incubation in PBS, the mixture was centrifuged at 14,000 rpm for 20 min for twice. Supernatant was removed. The amounts of free antigens in supernatant were estimated via FITC fluorescence intensities (for CSIINFEKL) or via UV absorbance at 280 nm (for Adpgk), and the loading amount of antigens was accordingly calculated.

### Physical characterization of iDR-NCs

For SEM, samples were deposited onto conductive glass, dried, and rinsed with diH_2_O. Dry samples were coated with Au (5 nm of thickness) and observed on an S-4800 SEM. TEM observation of dry iDR-NCs samples was also conducted. Preparation of NC cross section using FIB and the following SEM observation of NC cross section was conducted on a Tescan GAIA FIB/SEM at the Advanced Imaging and Microscopy Laboratory (AIMLAB). AFM samples were casted on mica substrate, dried, and washed with diH_2_O. AFM samples was observed in tapping-mode in air on a PicoForce Multimode AFM (Bruker, CA) equipped with a Nanoscope® V controller, a type E scanner head, and an OTESPA AFM cantilever. Results were analyzed using Nanoscope Software (ver. 7.3–8.15). The sizes of iDR-NCs suspended were measured using DLS on a Nanoparticle Analyzer (HORIBA Scientific, Tokyo, Japan). Bright field or fluorescence images of fluorophore-labeled iDR-NCs were taken on a Zeiss LSM 780 confocal microscope (Chesterfield, VA).

### Estimation of nucleic acid yields of RCR/RCT

iDR-NCs were treated with 5 mM EDTA, which chelated Mg^2+^ and dissolved MFs. DNA/RNA was purified by removing EDTA, Mg^2+^, and PPi^4−^ using centrifugation filtering (Millipore Ltd., Billerica, MA). The absorbance (260 nm) of the resulting DNA/RNA was determined on a Genesys 10 s UV-Vis spectrometer (ThermoFisher Scientific, Waltham, MA) and converted to the equivalent of CpG or shRNA.

### Biostability of iDR-NCs

iDR-NCs was treated with 5 U/mL DNase I (New England Biolabs, Ipswich, MA) at 37 °C for 1 h, followed by nuclease deactivation at 75 °C for 10 min. The stability of iDR-NCs against thermal denaturation was performed by heating iDR-NCs at specified temperature for 1 h. The morphologies of the above treated iDR-NCs were examined using SEM.

### Cell culture

RAW264.7 macrophages (ATCC) and MC38 cells (from Dr. Robert A. Seder Lab at NIAID) were cultured in DMEM medium with L-glutamine. DC2.4 cells (from Dr Jonathan W. Yewdell Lab at NIAID) were cultured in RPMI medium with L-glutamine. All medium was supplemented with 10% heat-inactivated FBS and 1% penicillin and streptomycin. Cells were cultured in a humidified atmosphere (5% CO_2_, 37 °C) in a Biosafety Level II culture room. All cell lines were confirmed as mycoplasma-negative.

### In vitro cell uptake of iDR-NCs

In vitro cell uptake was first studied using confocal laser scanning microscopy and flow cytometry. FITC-labeled AlbiCpG were incubated with RAW264.7 cells or DC2.4 cells, and stained with Lysotracker Red DND-99 (Life Technologies, Carlsbad, CA) and Hoechst 33342 (Life Technologies, Carlsbad, CA) according to manufacturer’s instruction. For confocal microscopy, cells were imaged on a Zeiss LSM 780 confocal microscope (Chesterfield, VA). For deconvolved confocal microscopy, cells were imaged on a Leica SP8 (Leica microsystems, Buffalo Grove, IL). Alternatively, flow cytometry was used to study the cell uptake using a BD Beckman Coulter flow cytometer (Brea, CA) and results were analyzed using FlowJo software V10 (TreeStar, Ashland, OR).

### Super-resolution imaging on instant SIM

In vitro intracellular uptake of iDR-NC-Alexa 555/CSIINFEK_(FITC)_L complexes was studied by super resolution in DCs, on a home-built instant SIM system^42^. The DC2.4 cells treated with iDR-NC-Alexa 555/CSIINFEK_(FITC)_L were washed with PBS and then immersed in HEPES buffer (pH 7.4) during imaging. FITC and Alexa 555 were excited using a 488-nm laser and a 561-nm laser, respectively. Acquired images were deconvoluted using a home-built software.

### RNA interference

siRNA or control were transfected using Lipofectamine2000 (Thermo Fisher Scientific) following manufacturer’s guidance. *Stat3* siRNA, primers for *Stat3* mRNA, Stattic, and p-STAT3 antibody were purchased from Santa Cruz Biotechnology.

### Concentrations of proinflammatory factors

Proinflammatory factors (TNFα, IL-6, IL-12p40) in cultured RAW264.7 or DC2.4 cells were quantified using ELISA according to manufacturers’ instructions. Cell culture medium from cells was collected at the specified time points post treatment respectively with iDR-NCs or the control regimes. Medium was diluted according to manufacturers’ instructions. Cell culture medium was then collected and centrifuged to remove any debris. The concentrations of cytokines in the culture medium of RAW264.7 or DC2.4 cells were assayed using ELISA (Life Technologies, Carlsbad, CA) as per manufacturer’s instructions.

### Expression levels of costimulatory factors on APCs

RAW264.7 macrophages and DC2.4 cells were studied by flow cytometry for the expression levels of costimulatory factor CD80. Cells were seeded into 24-well plate, and one day later treated with iDR-NCs or the corresponding control regimens at the specified concentrations, then cells were harvested and washed, resuspended in Dulbecco’s PBS and incubated with anti-CD80-PerCP (PeproTech, Rocky Hill, NJ) for 0.5 h on ice. Cells were then washed with Dulbecco’s PBS for three times for flow cytometry using a BD Beckman Coulter flow cytometer (Brea, CA). Results were analyzed using FlowJo Software (TreeStar, Ashland, OR).

### Animal studies

All animal work was conducted in appliance to the NIH Guide for the Care and Use of Animals under protocols approved by the NIH Clinical Center Animal Care and Use Committee. All studies on animals were evaluated in a blinding manner to investigators without prior knowledge of the specific treatments.

### In vivo delivery of iDR-NC/antigen into LN lymphocytes

Alexa 555 was labeled on iDR-NCs through an Alexa 555-conjugated primer. CSIINFEKL model antigen was modified with an FITC to be CSIINFEK_(FITC)_L. iDR-NC-Alexa 555/CSIINFEK_(FITC)_L complexes (in 50 μL reaction solution) were s.c.-injected into C57BL/6 mice at the tail base, and one day later, IN LNs were collected and smashed, treated with collagenase D (1 mg/mL. Sigma) and DNase I (10 U/mL. NEB) for 2 h at 37 °C to prepare single cells. Cells were filtered through a 40-μm strainer to remove tissue debris. The resulting single cells were stained with B220 (anti-B220-Alexa647, clone RA3-6B2, Biolegend) for B cells, F4/80 (anti-F4/80-APC, clone BM8, eBioscience) for macrophages, and CD11c (anti-CD11c-APC, clone N418, BioLegend) for DCs. Flow cytometry was conducted on a BD LSRFortessa X-50 flow cytometer. Results were analyzed in macrophage populations using FlowJo software (TreeStar, Ashland, OR) and GraphPad Prism 4 (La Jolla, CA).

### Dextramer and PD-1 staining on peripheral CD8^+^ T cells

C57BL/6 mice (6–8 weeks) were immunized with iDR-NC/Adpgk complexes (Adpgk: CGIPVHLELASMTNMELMSSIVHQQVFPT) on day 0 and day 14. Blood was collected from the above treated mice on day 21. Blood cells were enriched by centrifugation. Red blood cells were lysed using ACK lysis buffer for 5 min at room temperature. Blood clogs were removed using a plate filter. Cells were washed twice in PBS and cells were stained using DAPI in PBS buffer supplemented with anti-CD16/CD32 for 10 min at room temperature. Next, cells were added with dye-labeled staining cocktail including anti-CD8-APC-Cy7 (BioLegend), Dextramer-PE (Immudex), anti-PD-1-BV421 (BioLegend), according to manufacturer’s instructions. Cells were stained at room temperature for 30 min, washed, and 100 μL Cytofix was added into each well to resuspend cells for fixation at 4 °C for 20 min. Cells were then washed with Perm/Wash buffer, and resuspended for flow cytometric analysis on a BD LSRFortessa X-50 flow cytometer. Data were analyzed using FlowJo V10 and GraphPad Prism 4.

### Immune memory analysis

Mice were vaccinated as describe above. Peripheral blood was collected to analyze antigen-specific CD8^+^ T cells and memory T cells. Immune memory was analyzed by flow cytometric analysis of CD62L and CD44. Briefly, red blood cells were lysed, and blood cells were then collected, washed with PBS buffer, blocked with anti-CD16/CD32 for 10 min, followed by adding antibody cocktail that included anti-CD8-APC-Cy7 (clone 53–6.7, BioLegend), anti-CD44-PE-Cy5 (clone IM7, BD Bioscience), anti-CD62L-FITC (clone 30-F11, BD Bioscience), and dead cell-staining DAPI. Cells were stained at room temperature for 30 min, then washed, and 100 μL Cytofix (BD Biosciences) used to fix cells at 4 °C for 20 min. Cells were then washed with Perm/Wash buffer (BD Biosciences), and resuspended for flow cytometry on a BD LSRFortessa X-50 flow cytometer. Memory CD8^+^ T cells were analyzed as following: central memory cells (CD44^+^CD62L^high^), effector memory cells (CD44^+^CD62L^−^), and naive CD8^+^ T cells (CD44^-^CD62L^+^).

### Tumor model and combination cancer immunotherapy

C57BL/6 mice (6–8 weeks) were *i*.*v*. injected with MC38 cells (1 × 10^5^). On day10, mice were randomly divided into groups (6–7 per group) that were respectively vaccinated with (1) PBS, (2) CpG and Adpgk, and (3) iDR-NCs and Adpgk, by subcutaneous injection in 50 μL at the base of tail on day10, day 16, and day 22 post tumor inoculation. Tumor burden was quantified at the end of study using FDG radiotracer. Specifically, mice were anesthetized for 30 min using isoflurane/O^2^ (2% v/v) before injection. Anesthetized mice were injected *i*.*p*. with FDG (3.7 MBq per mouse) in PBS (100 μL). Mice continued to be anesthetized for 1 h, and then, one mice from each group was randomly picked to be scanned for PET/CT on a nanoScan PET/CT scanner (Mediso Medical Imaging Systems). Meanwhile, mice were euthanized and organs of interest were resected, followed by measuring ^18^F radioactivity on a gamma-counter (Wallac Wizard 1480, PerkinElmer). The radioactivity in organs was converted to calculate the percentages of the %ID and %ID per g in organs of interest. Results were analyzed using GraphPad Prism 4 (La Jolla, CA).

### Data availability

The authors declare that the data supporting the findings of this study are available within the article and its Supplementary Information Files or from the corresponding authors on request.

## Electronic supplementary material

Supplementary Information

Description of Additional Supplementary Files

Supplementary Movie 1
