# Supplementary material for: Intertwining DNA-RNA nanocapsules loaded with tumor neoantigens as synergistic nanovaccines for cancer immunotherapy
Source: Nat Commun. 2017 Nov 14;8:1482. doi: 10.1038/s41467-017-01386-7 (PMC5684198; doi:10.1038/s41467-017-01386-7)
Supplement: Supplementary file 1 — Supplementary Information [file 41467_2017_1386_MOESM1_ESM.pdf]

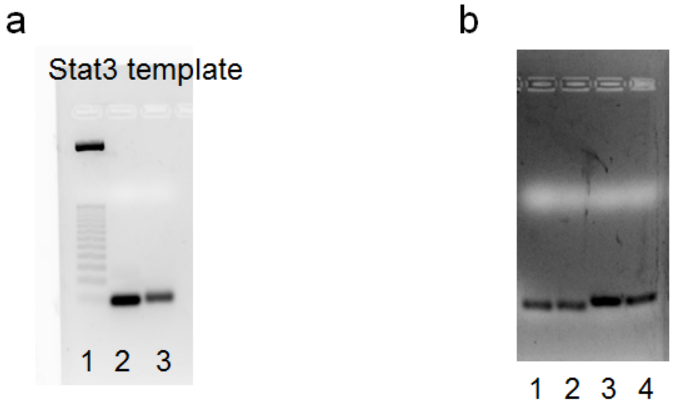

a

1. 25bp Ladder
2. Unligated
3. Ligated

b

1. Control circular DNA
2. Control circular DNA + ExoI and Exo III
3. Circular stat3 template
4. Circular stat3 template + ExoI and Exo III

Supplementary Figure 1. Images of agarose gel electrophoresis verifying the circularization of template for Stat3 shRNA.

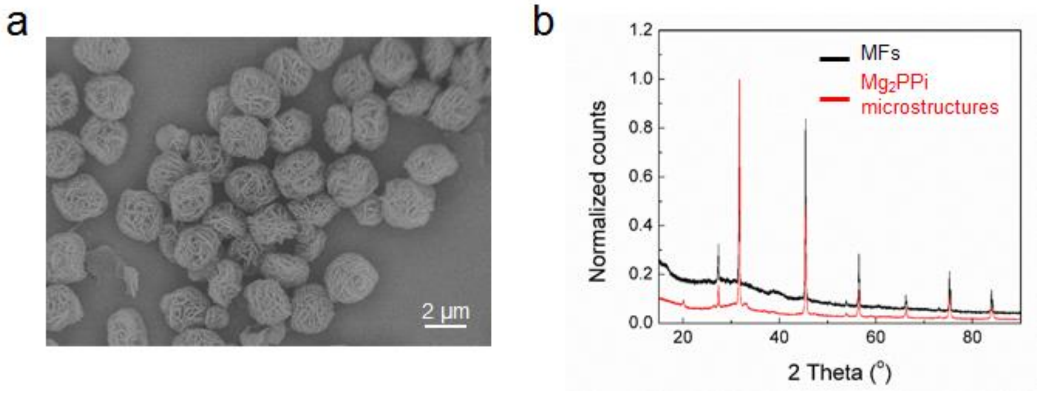

Supplementary Figure 2. Characterization of DNA-RNA MFs. (a) An SEM image showing pure Mg_2PPi nanostructures formed by mixing Mg^{2+} (16 mM) with PPi^{4-} (5 mM). (b) XRD graphs of DNA-RNA microflowers (MFs) generated from mixed RCT/RCR, and Mg2PPi microstrcutures.

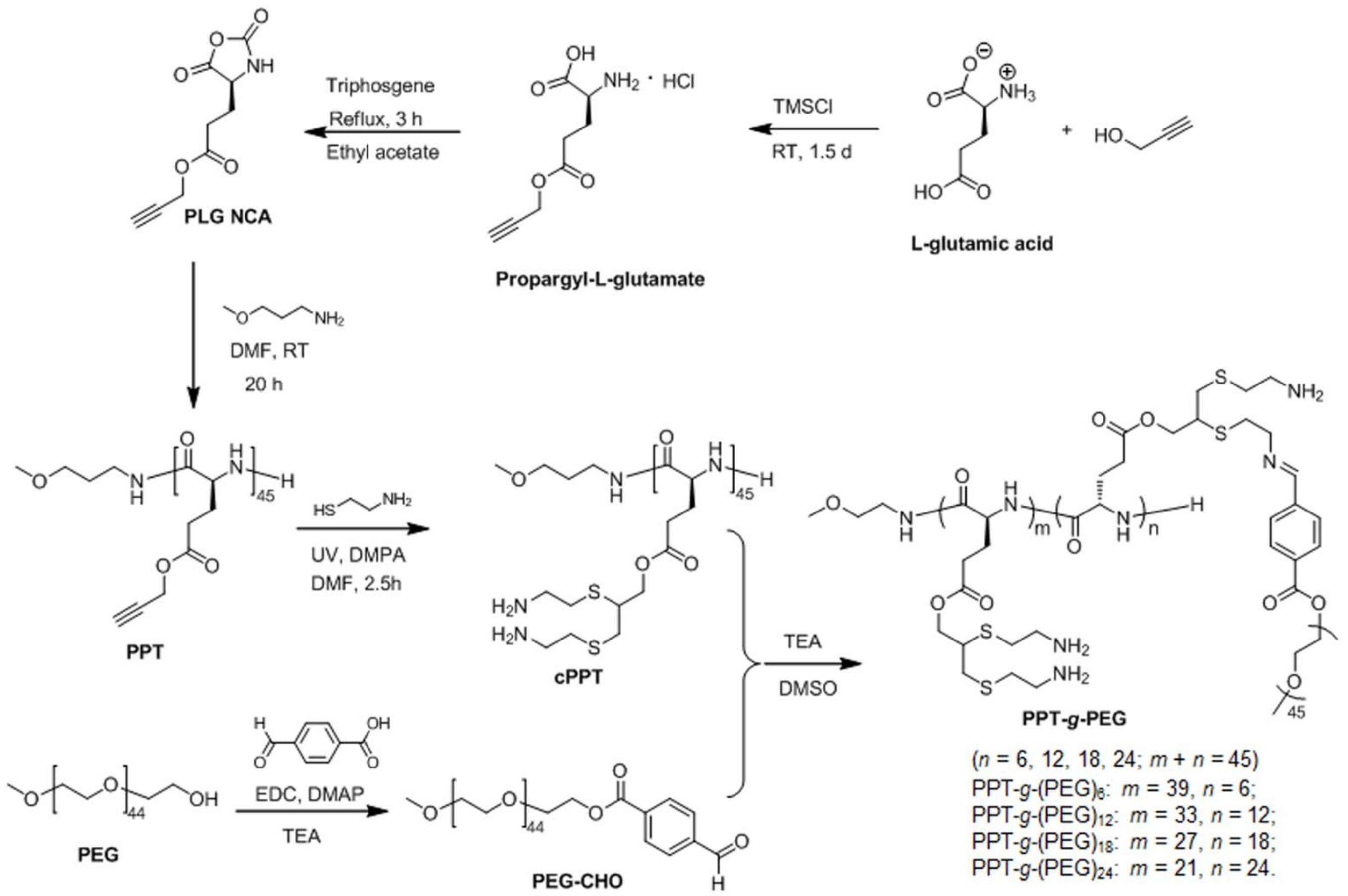

Supplementary Figure 3. Synthesis scheme of multifunctional and biocompatible PPT-*g*-PEG derivatives with acid-responsively cleavable PEG.

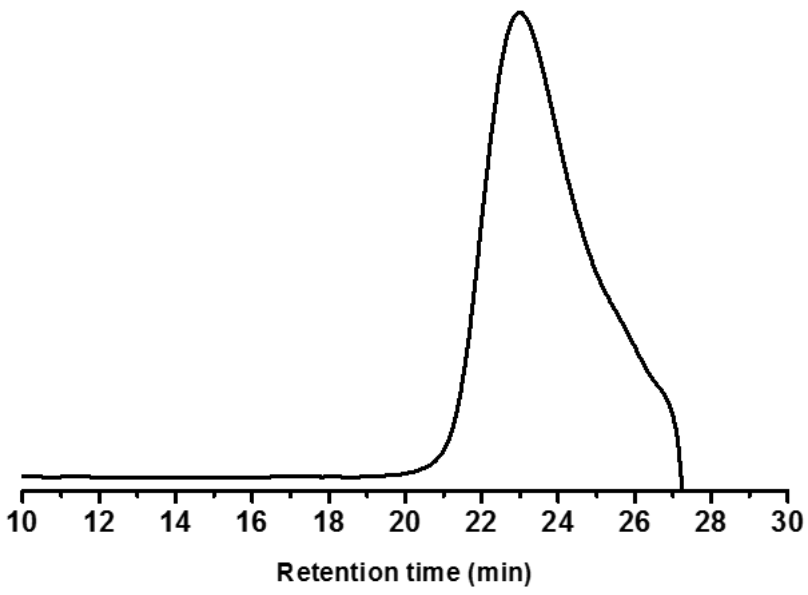

Supplementary Figure 4. Gel permeation chromatography trace of PPT. M_n 15145 Da, M_w 18691Da, PDI: 1.23, using poly(methyl methacrylate)s as calibration standards.

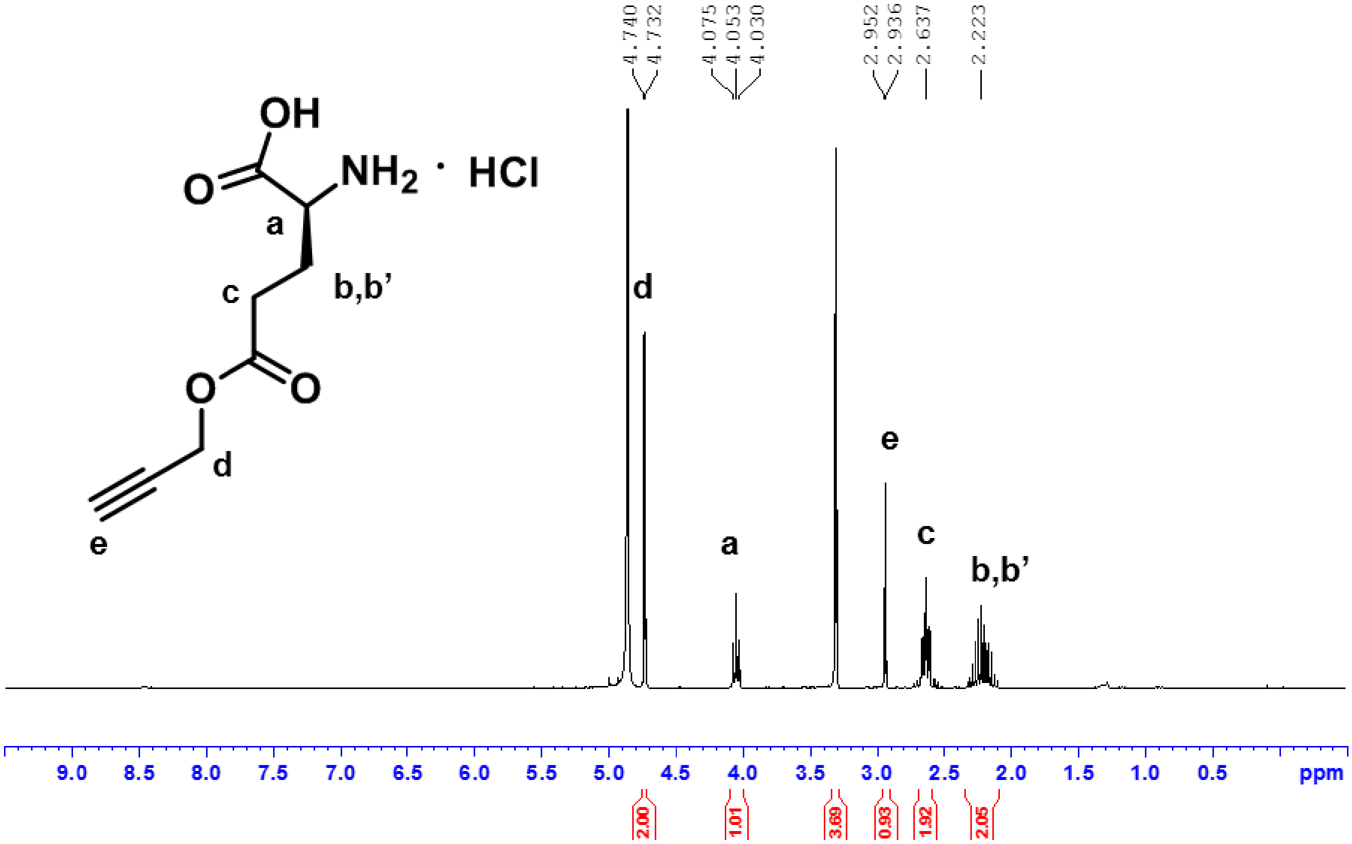

Supplementary Figure 5. ^{1}**H NMR of Propargyl-L-glutamate in MeOD**.

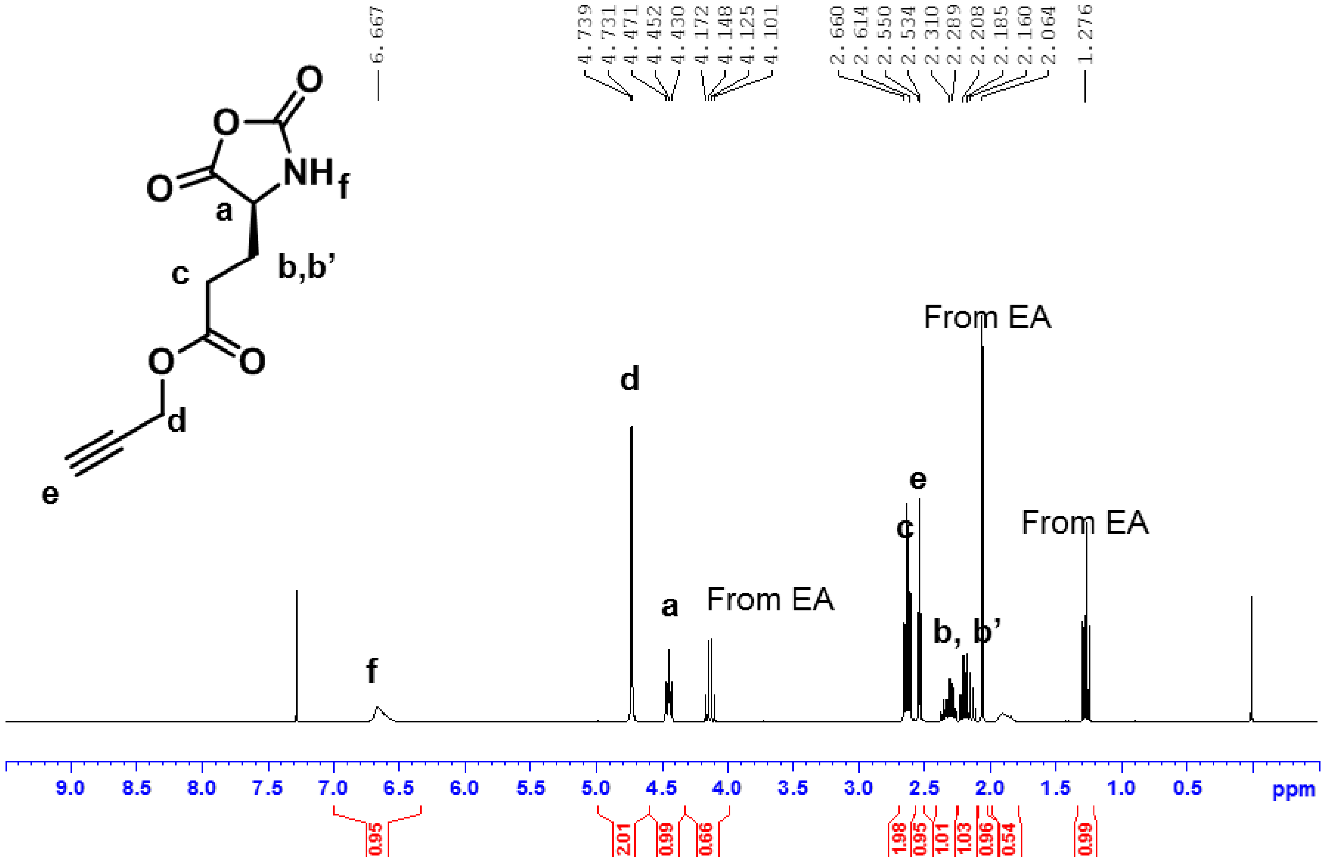

Supplementary Figure 6. ^{1}H NMR of PLG NCA in $CDCl_3$.

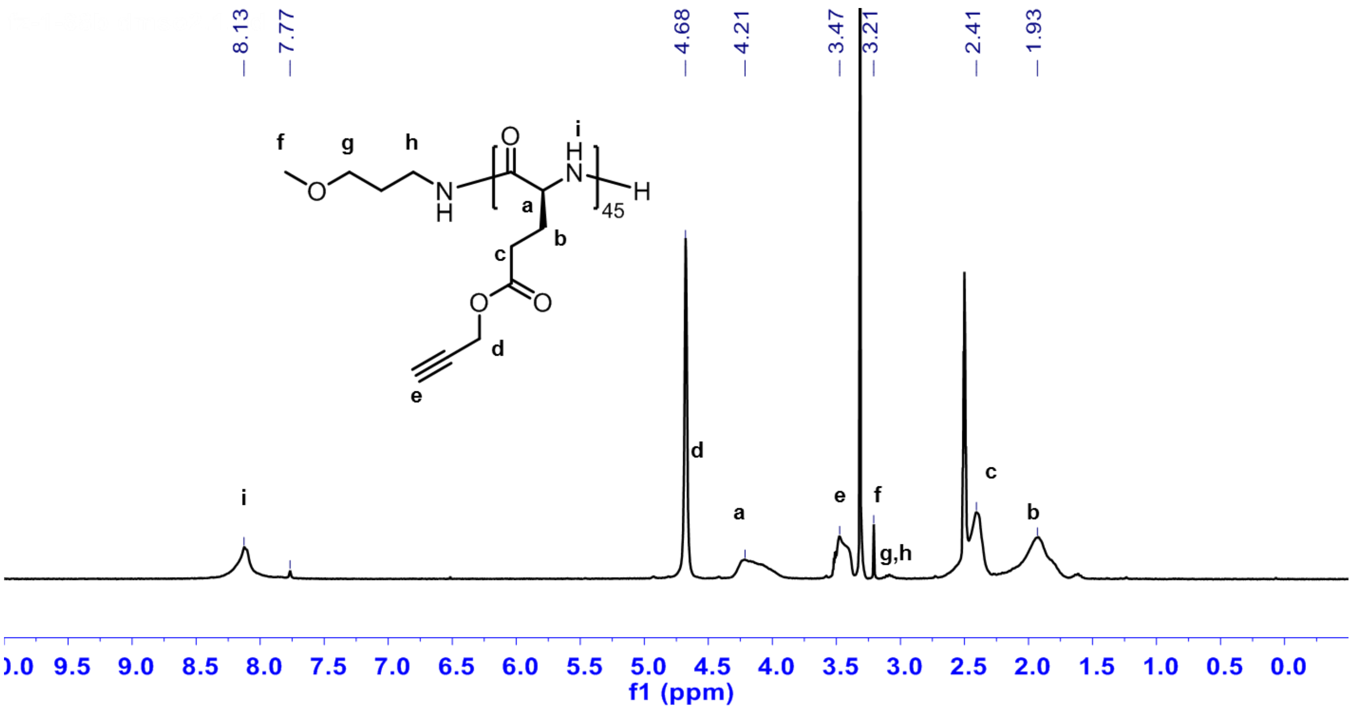

Supplementary Figure 7. ^{1}H NMR of PPT_{45} in DMSO-d_6.

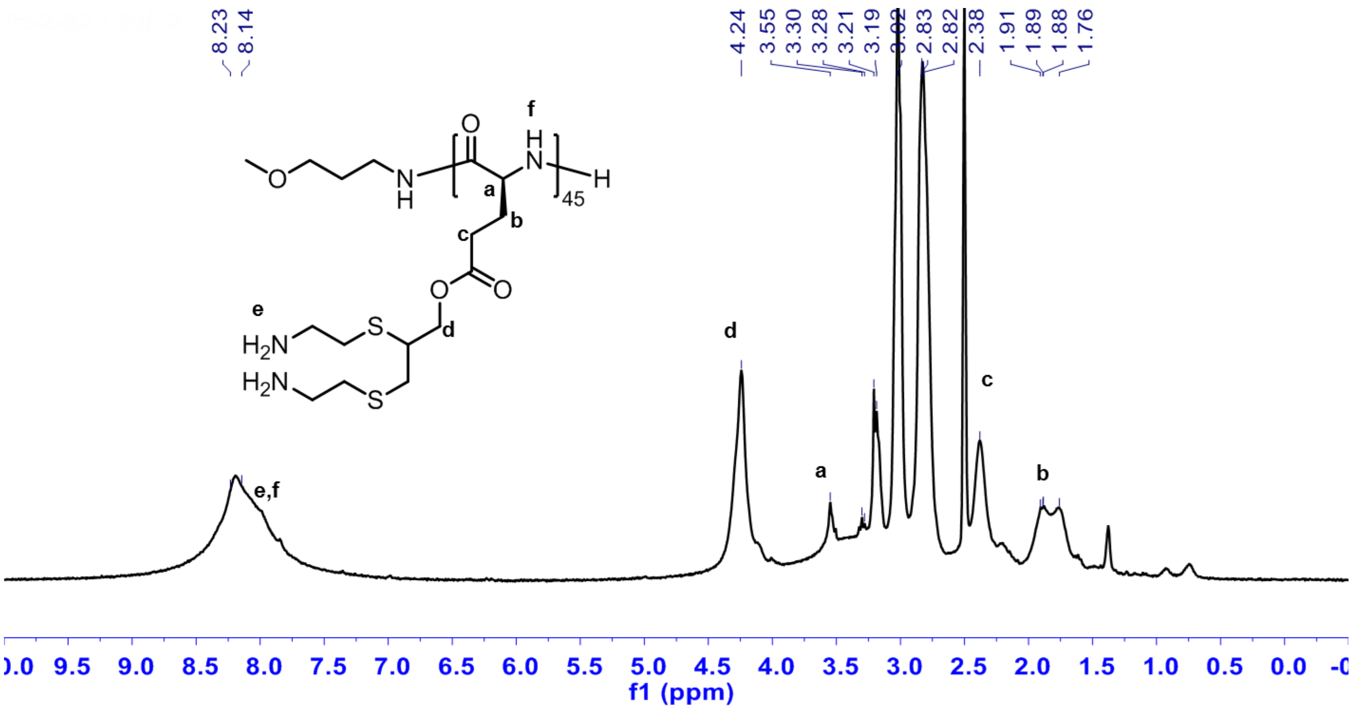

Supplementary Figure 8. ^{1}H NMR of cPPT$_{45}$ in DMSO-d$_6$.

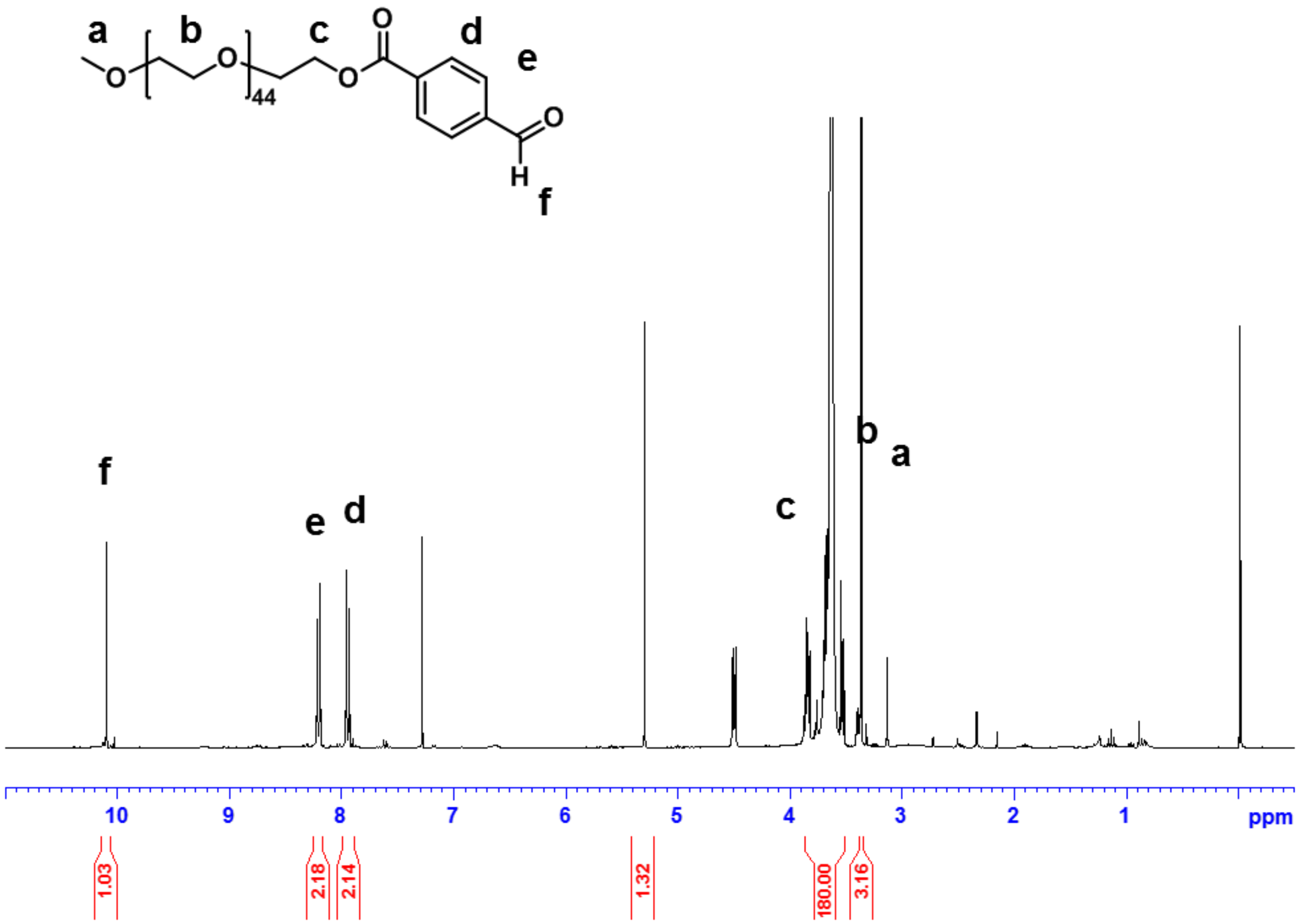

Supplementary Figure 9. ^{1}H NMR of PEG-CHO in $CDCl_3$.

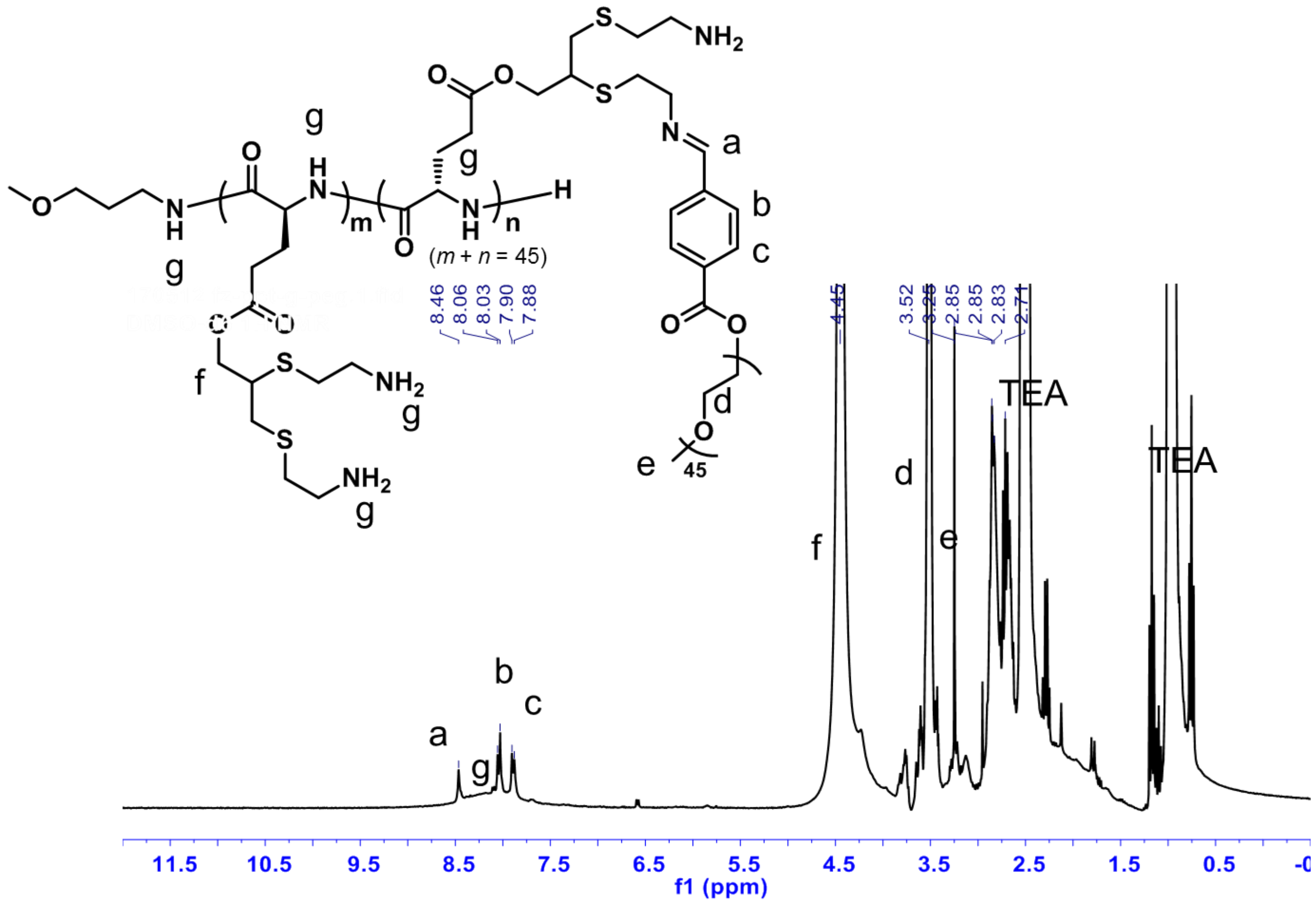

Supplementary Figure 10. ^{1}H NMR of PPT-*g*-PEG in DMSO-d$_6$. The aldehyde proton at δ 10.12 disappeared completely, but a new peak appeared at δ 8.46, indicating the complete conversion of CHO to Schiff base.

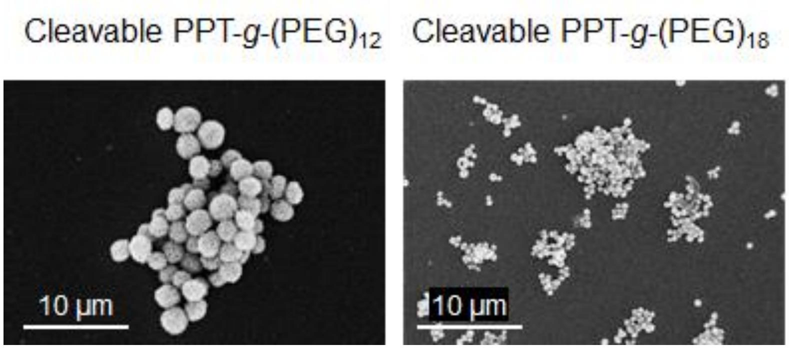

Supplementary Figure 11. SEM images showing the morphology of residual DNA-RNA microflowers after incubation with PPT-*g*-$(PEG)_{12}$ and PPT-*g*-$(PEG)_{18}$ polymers for 48 h. This indicates the incomplete shrinkage under these conditions.

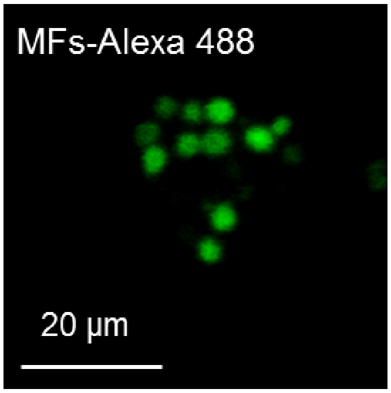

Supplementary Figure 12. Fluorescence image of DNA-RNA microflowers (MFs) labeled with Alexa 488 through Alexa 488-labeled DNA primer.

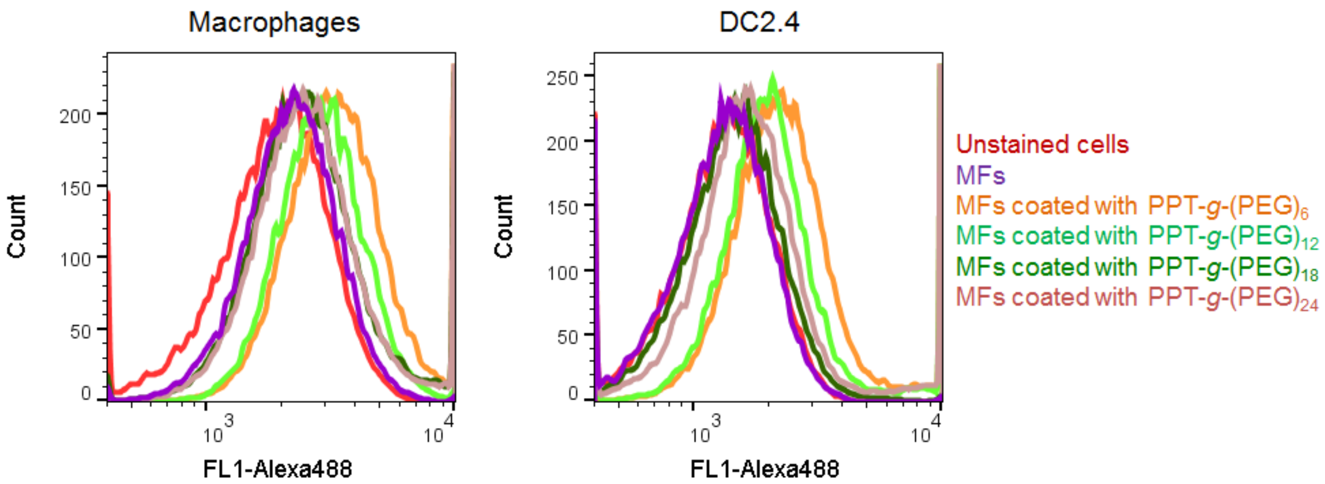

Supplementary Figure 13. Flow cytometry results showing the ability of RAW264.7 cells and DC2.4 cells to internalize microflowers (MFs) treated with a series of PPT-*g*-PEG copolymers for shrinkage. Alexa 488 was labeled on the original MFs via DNA primers used for DNA RCR.

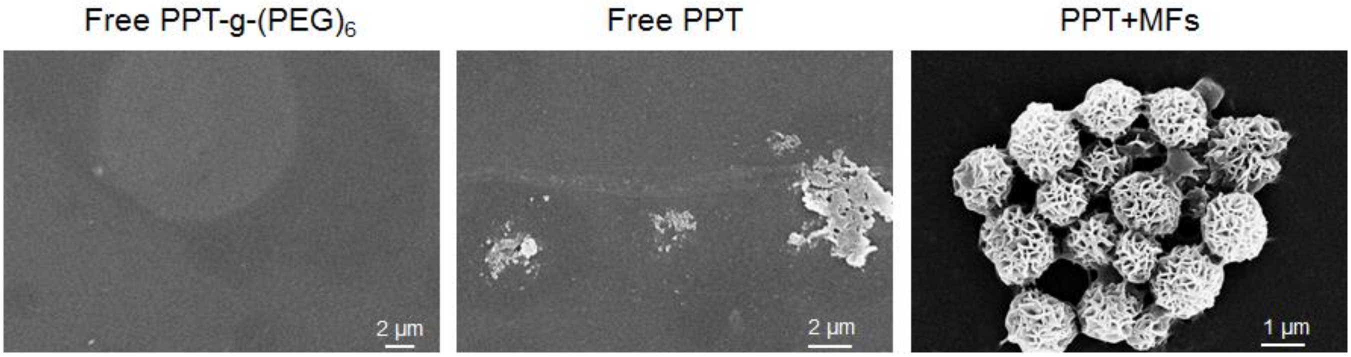

Supplementary Figure 14. SEM images showing the morphology of free PPT-*g*-$(PEG)_6$ polymers, free PPT, and DNA-RNA microflowers mixed in PPT solutions.

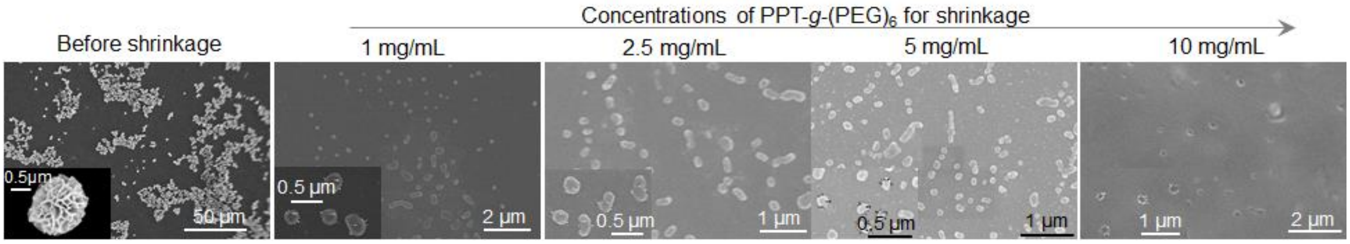

Supplementary Figure 15. SEM images showing the products before and after incubation of DNA-RNA microflowers with increasing concentrations of PPT-*g*-$(PEG)_6$ for 48 h.

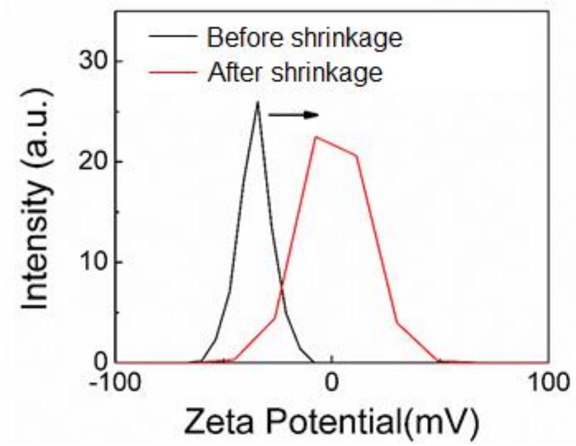

Supplementary Figure 16. Zeta potential of DNA-RNA microflowers before (-35.3 mV) and after (1.5 mV) shrinking using PPT-*g*-$(PEG)_6$ for 48 h.

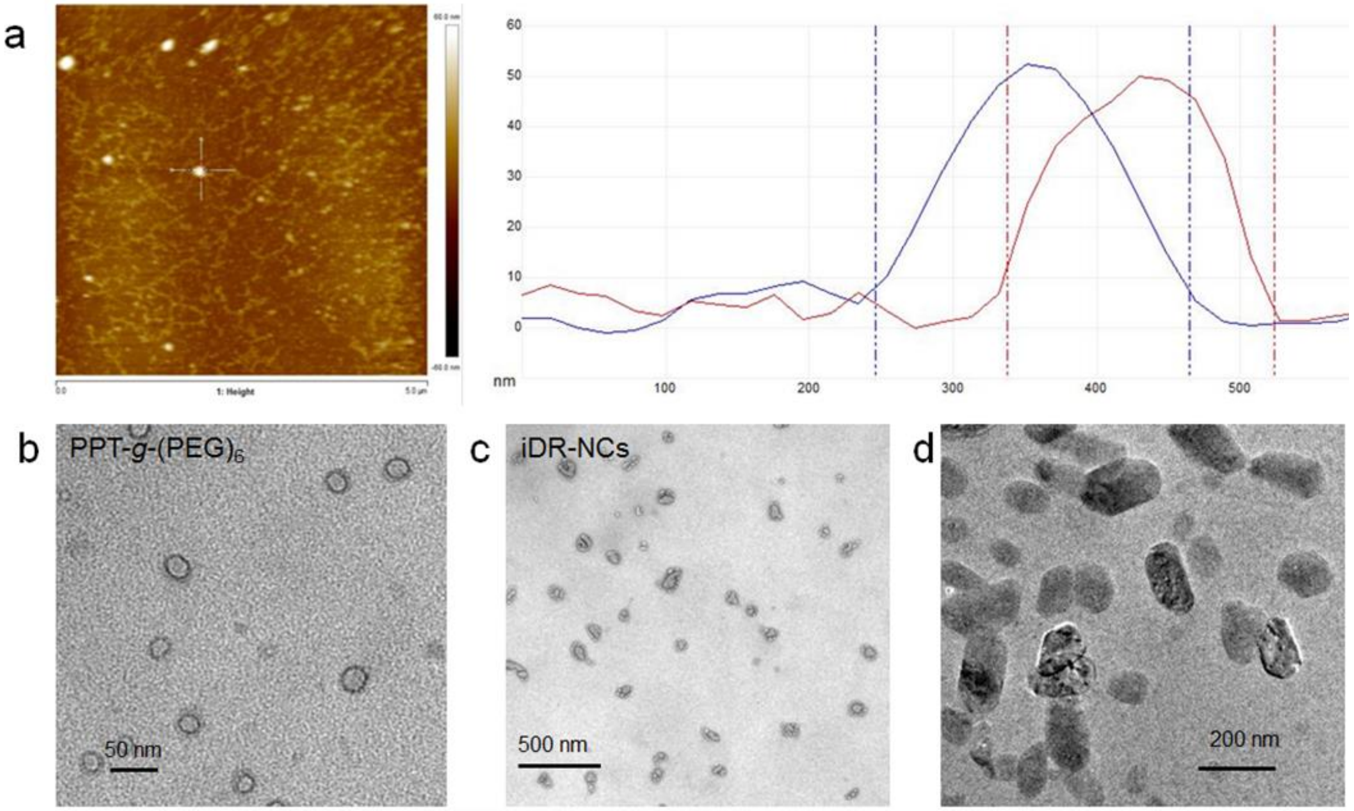

Supplementary Figure 17. (a) AFM images (left) and the corresponding dimension measurement results (right) of iDR-NCs. The red lines in AFM images marked the location of dimension measurement. (b) A TEM image showing the morphologies of small nanoparticles formed by PPT-*g*-(PEG)$_6$ polymers (c) TEM images showing the morphologies of iDR-NCs shrunk by PPT-*g*-(PEG)$_6$. (d) A HR-TEM image showing the morphologies of iDR-NCs shrunk by PPT-*g*-(PEG)$_6$.

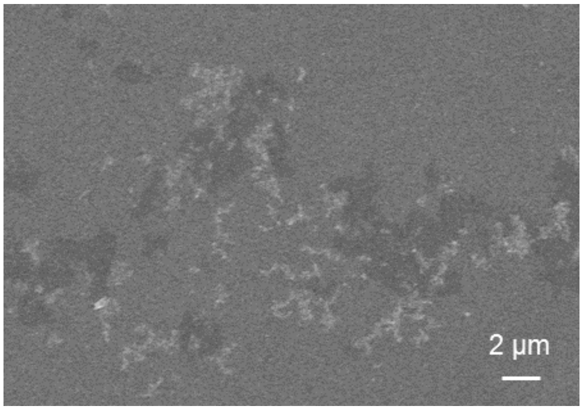

Supplementary Figure 18. An SEM image showing that DNA-RNA MFs were dissolved by treating with EDTA (5 mM) for 30 min.

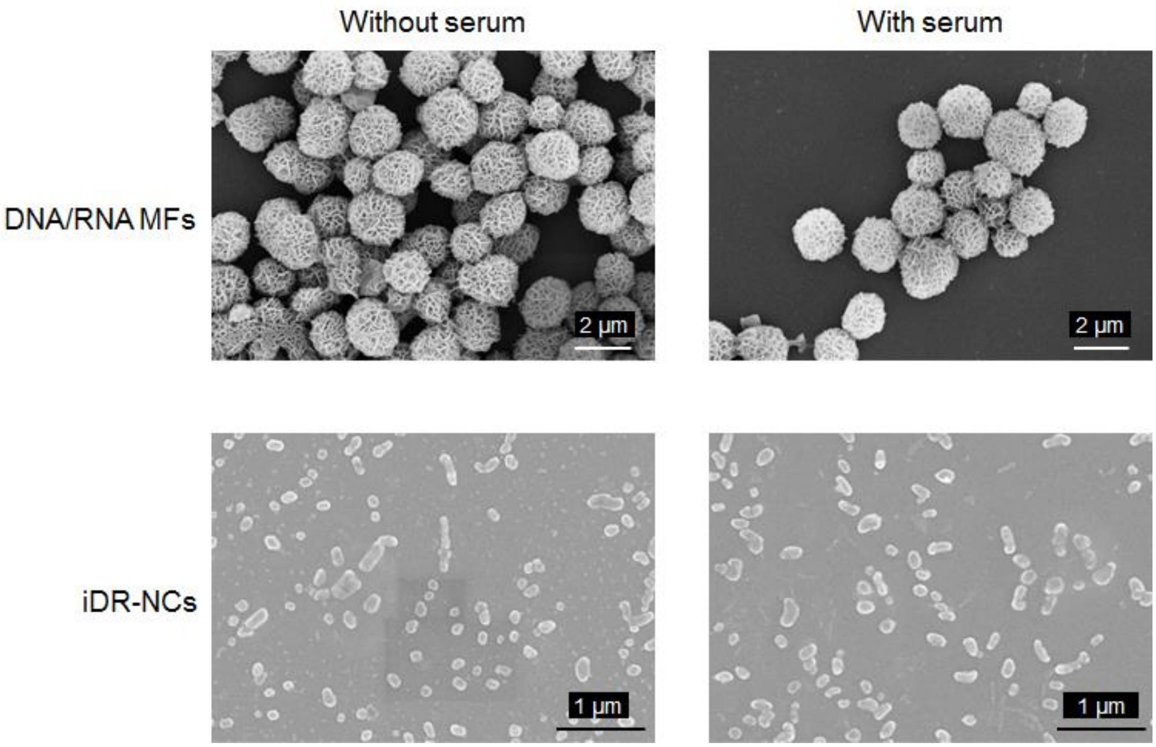

Supplementary Figure 19. SEM images showing the comparison of the morphhologies of DNA-RNA MFs (upper panel) and iDR-NCs (lower panel) with or without incubation in serum. The undetectable changes upon incubation in serum demonstrates the high biostability of iDR-NCs.

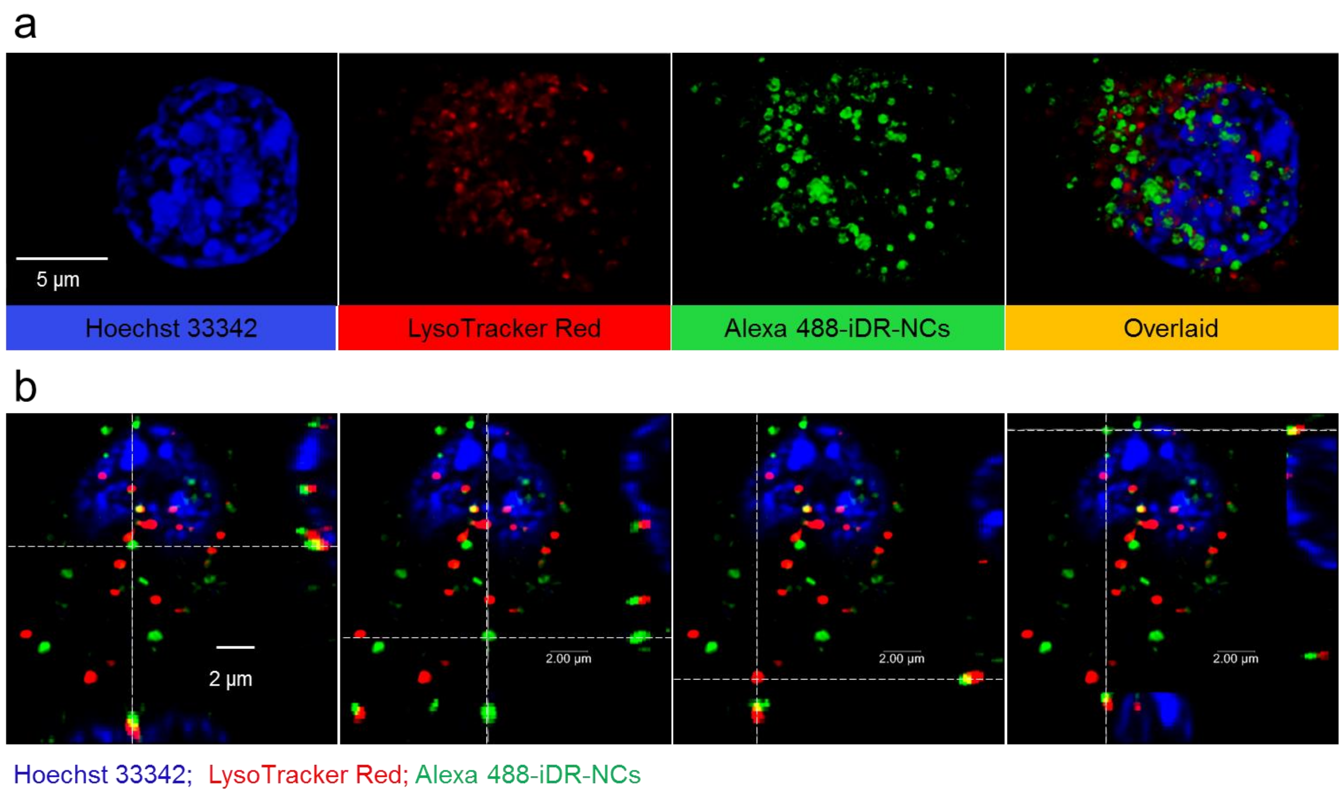

Supplementary Figure 20. Super-resolution confocal microscopy images displaying that Alexa 488-labeled iDR-NCs were efficiently delivered into DC2.4 cells and resided in the endolysosome after incubation for 3 h. (**a**) One DC2.4 cell showing individual or overlaid signals. (**b**) A panel of cross-section images showing the fluorescence signals at 3 different dimensions. The 1st, 3rd, and 4th images showed the presence of iDR-NCs in endolysosome, and the 2nd image showed the presence of iDR-NCs in cytosol. The endolysosome was stained by Lysotracker Red DND-99, and nucleus was stained by Hoechst 33342.

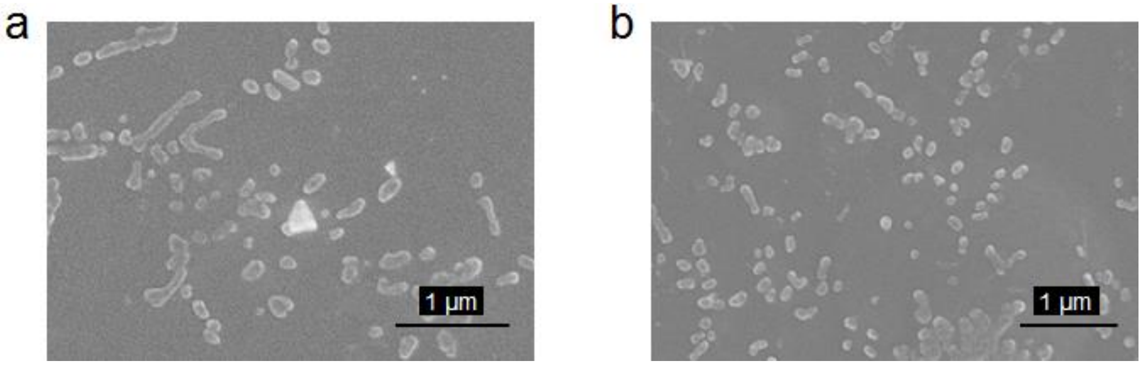

Supplementary Figure 21. TEM images of iDR-NC/CSIINFEKL complexes (**a**) and iDR-NC/Adpgk complexes (**b**).

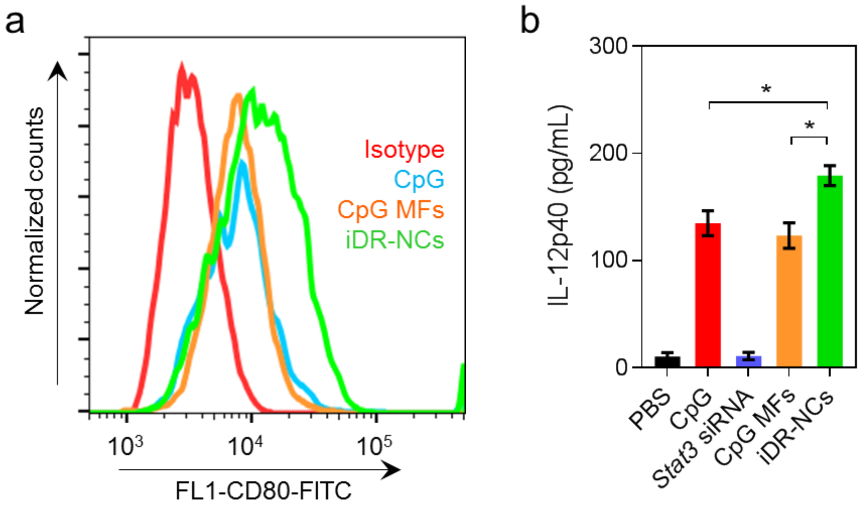

Supplementary Figure 22. Immunostimulation of iDR-NCs in RAW264.7 macrophages. (a) Flow cytometry results showing that iDR-NC (100 nM equivalent CpG for 24 h) elevated the expression of costimulatory factors CD80 in RAW264.7 macrophages. (b) ELISA results suggest iDR-NCs induced RAW264.7 macrophages to secret significantly more proinflammatory cytokines IL-12p40 than CpG or CpG microflowers (MFs), after treating cells at the concentrations of 100 nM equivalent CpG for 24 h. As a control, *stat3* siRNA were not immunostimulatory.

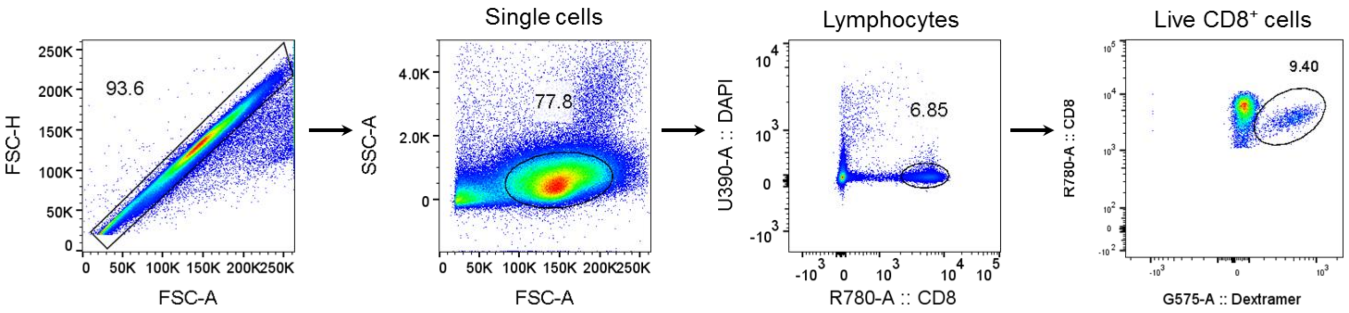

Supplementary Figure 23. Gating tree used for flow cytometric analysis of antigen-specific CD8$^+$ T cells in dextramer staining studies.

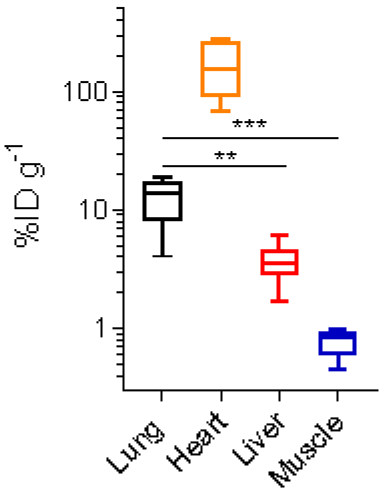

Supplementary Figure 24. Percent of injected dose of FDG per gram of tissue (%ID/g) in organs of interest on day 40 post immunotherapy of MC38 tumor.

Supplementary Table 1. DNA sequences. CpG (CpG 1826) had phosphorothioate backbone unless denoted otherwise, and all other DNA had phosphodiester backbone. Purple sequences: CpG or CpG analogs; Red sequences: GpC or GpC analogs; Underlined sequence: complementary sequences between primers and the corresponding templates; Shaded sequence: CpG or GpC dinucleotide.

	Sequences (5'-3')
**CpG**	TCCATGACGTTCCTGACGTT
**GpC**	TCCATGAGCTTCCTGAGCTT
**Primer for NCs**	ACGTTCCTGACGTTTTTCAGCGTGACTTTTCCATGACGTTCC
**Template for NCs**	5'-phosphate-CGCTGAAAAACGTCAGGAACGTCATGGAAAAAAACGTCAGGAACGTCATGGAAAAAAACGTCAGGAACGTCATGGAAAAGTCA
**Primer for GpC-NFs**	AGCTTCCTGAGCTTTTTCAGCGTGACTTTTCCATGAGCTTCC
**Template for GpC-NFs**	5'-phosphate-CGCTGAAAAAGCTCAGGAAGCTCATGGAAAAAAAGCTCAGGAAGCTCATGGAAAAAAAGCTCAGGAAGCTCATGGAAAAGTCA
**Stat3 siRNA sense strand**	CAGGGUGUCAGAUCACAUGGGCUAA
**Stat3 siRNA antisense strand**	UUAGCCCAUGUGAUCUGACACCCUGAA
**Stat3 linear template**	5'-phosphate-TGACACCCTGTGACAGGAAGTTCAGGGTGTCAGATCACATGGGCTAATTGTTTTAGCCCATGTGATC

Supplementary Table 2. Comparison of RCR and RCT.

	RCR	RCT
**Product**	DNA	RNA
**Temperature (ºC)**	30	37
**Enzyme**	Phi29 DNA polymerase	T7 RNA polymerase
**Buffer composition**	50 mM Tris-HCl<br>10 mM $MgCl_2$<br>10 mM (NH4)2SO4<br>4 mM dithiothreitol	40 mM Tris-HCl<br>6 mM $MgCl_2$<br>2 mM spermidine<br>1 mM dithiothreitol
**Primer/promotor?**	Often rely on a separate primer DNA	Not always rely on a separate primer/promotor DNA
