# Supplementary material for: Intertwining DNA-RNA nanocapsules loaded with tumor neoantigens as synergistic nanovaccines for cancer immunotherapy
Source: Nat Commun. 2017 Nov 14;8:1482. doi: 10.1038/s41467-017-01386-7 (PMC5684198; doi:10.1038/s41467-017-01386-7)
Supplement: Supplementary file 2 — Description of Additional Supplementary Files [file 41467_2017_1386_MOESM2_ESM.pdf]

Description of Additional Supplementary Files

File Name: Supplementary Movie 1
Description: 3D reconstructed super-resolutoin confocal microscopy of one DC that efficiently internalized Alexa488-labeled iDR-NCs.
